# Interactions of Gut Microbiota, Endotoxemia, Immune Function, and Diet in Exertional Heatstroke

**DOI:** 10.1155/2018/5724575

**Published:** 2018-04-16

**Authors:** Lawrence E. Armstrong, Elaine C. Lee, Elizabeth M. Armstrong

**Affiliations:** ^1^Department of Kinesiology, Human Performance Laboratory, University of Connecticut, Storrs, CT 06269-1110, USA; ^2^Oncology Nutrition, Hartford Hospital Cancer Institute, Hartford, CT 06102, USA

## Abstract

Exertional heatstroke (EHS) is a medical emergency that cannot be predicted, requires immediate whole-body cooling to reduce elevated internal body temperature, and is influenced by numerous host and environmental factors. Widely accepted predisposing factors (PDF) include prolonged or intense exercise, lack of heat acclimatization, sleep deprivation, dehydration, diet, alcohol abuse, drug use, chronic inflammation, febrile illness, older age, and nonsteroidal anti-inflammatory drug use. The present review links these factors to the human intestinal microbiota (IM) and diet, which previously have not been appreciated as PDF. This review also describes plausible mechanisms by which these PDF lead to EHS: endotoxemia resulting from elevated plasma lipopolysaccharide (i.e., a structural component of the outer membrane of Gram-negative bacteria) and tissue injury from oxygen free radicals. We propose that recognizing the lifestyle and host factors which are influenced by intestine-microbial interactions, and modifying habitual dietary patterns to alter the IM ecosystem, will encourage efficient immune function, optimize the intestinal epithelial barrier, and reduce EHS morbidity and mortality.

## 1. Introduction

Exertional heatstroke (EHS), resulting from prolonged exercise-induced hyperthermia, is one of the few disorders that disables young, healthy, physically fit individuals [1]. When the metabolic heat produced by muscle during exercise or labor exceeds body heat dissipation to the surrounding environment, internal body temperature rises to a level that disrupts organ function. Clinically, EHS is a medical emergency defined by hyperthermia (internal body temperature > 40°C) associated with central nervous system and multiple-organ dysfunction; thus, EHS is distinguished from other exertional heat illnesses (e.g., exertional heat exhaustion, heat syncope, and heat cramps) and from the physiological responses which result from exercise-heat stress (i.e., heat strain) [2, 3]. Although numerous PDF for EHS have been described (Table 1), they all potentiate hyperthermia in deep body tissues [1, 4]. In a report regarding EHS in a large military cohort, Abriat et al. [5] stated, “…determination of the factors contributing to [EHS] recurrence is urgently needed….” This call for action arose from their observation that 15% of EHS patients experienced a similar event at a later date. Equally troubling data were published by Leon and Helwig [6] regarding longevity of former military EHS patients. When compared to patients with a non-heat-related illness, former EHS patients had double the mortality from cardiovascular, kidney, and liver failure within 30 years of hospitalization. Unfortunately, the cause(s) of recurrent EHS and increased morbidity and mortality are unknown.

EHS cannot be predicted and often strikes athletes, laborers, and soldiers during activities that they have performed previously, in similar environmental conditions, at the same exercise intensity-duration, and while wearing similar clothing/gear. Thus understanding the predisposing factors (PDF) for EHS, and taking proactive measures to counteract those factors, reduces the risk of injury or death. Previously, members of our laboratory proposed the intestinal microbiota (IM) as a predisposing factor that deserves greater consideration in the etiology of EHS [7]. The present article again proposes the IM as a key factor in the etiology of EHS but further describes the interactions of the IM with intestinal ultrastructure, gut physiological functions, the immune system, illness, diet, exercise, and personal characteristics. The role of endotoxemia is emphasized. This article also recommends steps to reduce EHS morbidity and mortality by encouraging efficient metabolic and immune system function.

## 2. Characteristics of the IM

**Figure 1:**
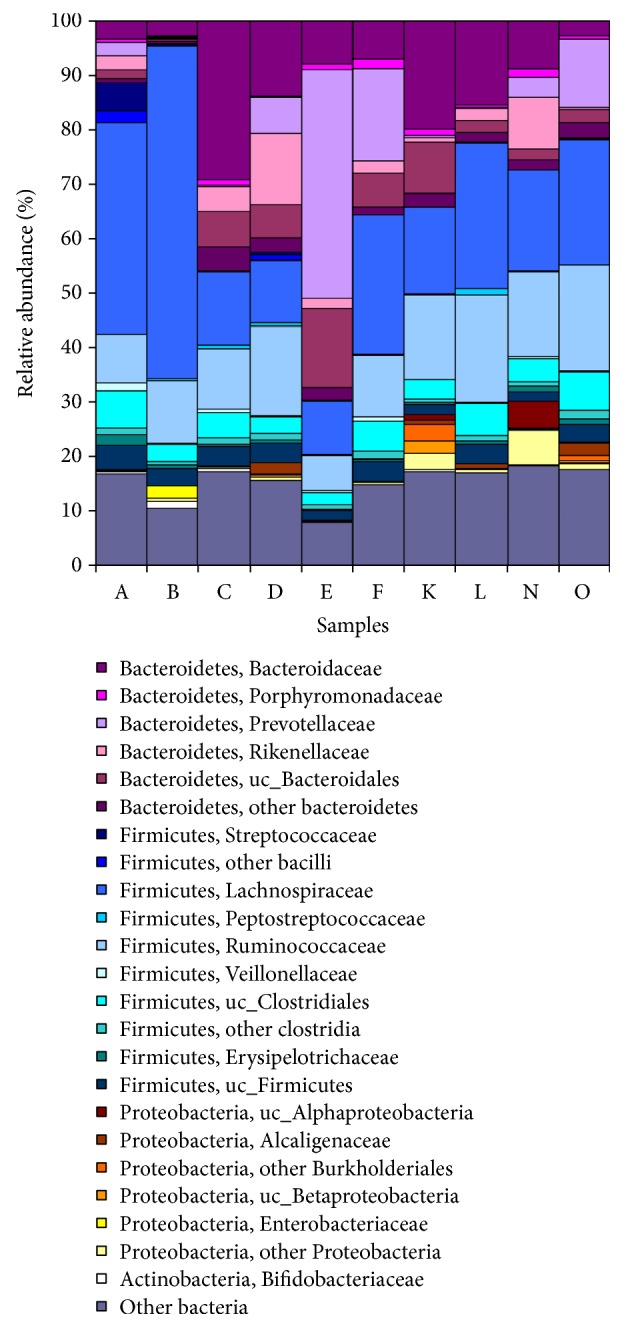
The relative abundance of the most common bacterial phyla in the active IM, analyzed in fecal samples of 10 healthy adults (A–O). Reprinted from Gosalbes et al. [349].

**Figure 2:**
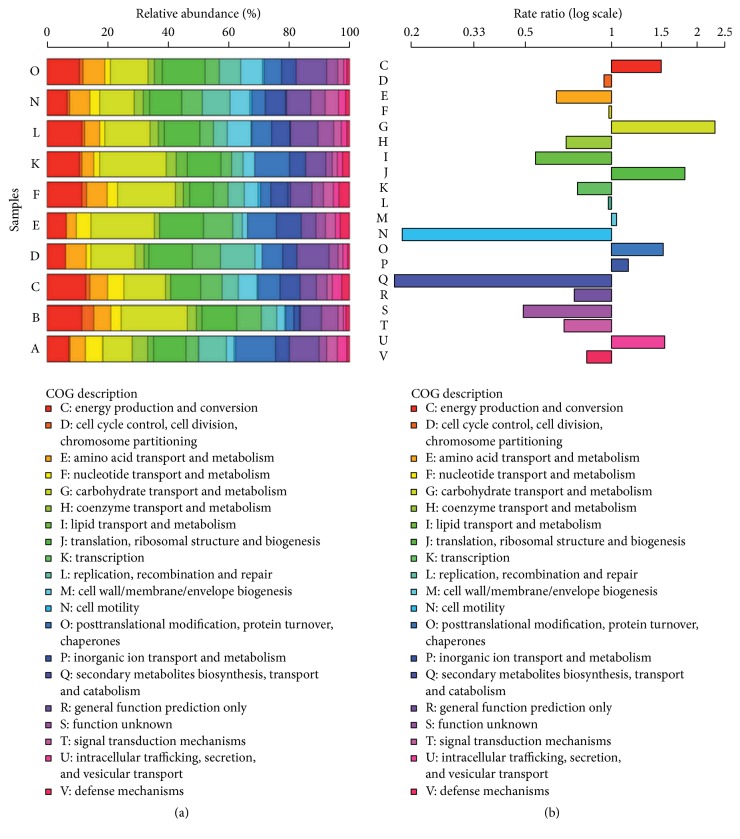
The relative abundance (%) of bacterial processes in the IM of 10 healthy adults (labelled A–O, Panel A, *y*-axis). Sequence data from 10 individuals was searched against a database (gCOGdb) of completely sequenced bacterial genomes (*n*  = 1,012; National Center for Biotechnology Information, 2009) and assigned to Clusters of Orthologous Groups (COG) categories, which indicate likely protein function. Data suggest that carbohydrate and lipid metabolism, energy production, and synthesis of cellular components are the main functions of gut microbiota in these individuals. The rate ratio value (Panel B) was calculated to measure whether COG patterns observed in the 10 individuals were likely due to biases in the existing reference database (gCOGdb). Rate ratios ≠ 1.0 indicate that some categories are over- or underrepresented in the sample of 10 individuals compared to the gCOGdb; these processes likely represent real differences in processes among the 10 individual IM evaluated, above and beyond artifact that might be present in the distribution of COG categories in the general gCOGdb. Reprinted from Gosalbes et al. [349].

Population studies indicate that 300–500 different species of bacteria constitute the majority of microorganisms in the human gut [8]. When rare, low-abundance, uncultivated or unclassified bacteria are considered, the healthy human gut likely contains more than 1000 species [9]. The Bacteroidetes (genera* Bacteroides* and* Prevotella*) and the Firmicutes (genera* Clostridium*,* Eubacterium*, and* Ruminococcus*) (Figure 1; Table 2) account for more than 90% of the IM population [10, 11]. Although the exact mechanisms and scope of influence are unknown, primarily because this ecosystem remains incompletely characterized and its diversity poorly defined, the human IM apparently exerts a broad range of health-related effects [12]. These include intestinal development during infancy and malnutrition, allergies, appetite control, energy balance, and pathology, or prevention of metabolic diseases such as obesity, diabetes, inflammatory bowel diseases, neurological disorders, cancers, and cardiovascular diseases [10, 12–14]. The IM also has been implicated in the development of central and peripheral neural processes (i.e., the brain-gut-enteric microbiota axis), as well as the central response to stress via the hypothalamic-pituitary-adrenal axis [11]. These wide ranging effects on human health have prompted many investigators to consider the role of IM diversity. Individuals with a greater IM species diversity apparently have a greater repertoire of microbial metabolic functions (Figure 2), a gut microbiome which is more functionally robust and which theoretically allows them to cope with homeostatic disruptions more effectively than individuals with less IM species diversity [9, 15]. For example, greater bacterial diversity was shown to correspond with better nutritional status, fewer comorbidities, and greater overall health in a cohort of elderly individuals [16]. Thus, it is generally agreed upon that the characteristics of a healthy microbiota include community stability and increased species diversity [17]. Theoretically, greater IM diversity provides the host with a wider repertoire of bacterial processes (i.e., metabolism, nutrient transport, energy production, cell signaling, reproduction, and growth) to maintain IM homeostasis and health (Figure 2).

## 3. Diet and Exercise Alter IM Abundance and Diversity

Specific microbe classes (i.e., which enhance metabolism, resilience to infection/inflammation, resistance to autoimmunity, and endocrine signaling) have been considered to be important in human health. Examples of bacterial taxa that have been associated with health and effective gastrointestinal function include* Bacteroides, Bifidobacterium*,* Eubacterium, Faecalibacterium, Lactobacillus,* and* Roseburia* [15]. However, multiple factors can alter the ratio of bacterial families across time, including disease and diet [13]. For example, the ratio of two major genera of gut bacteria,* Prevotella* and* Bacteroides*, were different in a community that consumed a high-fiber diet (e.g., Africa, Malawi, and Venezuela) dominated by maize, cassava, and other plant polysaccharides, versus one that consumed a diet rich in animal protein and saturated fats (e.g., USA and Europe). Also, as part of a dietary intervention study, Russell and colleagues [18] provided 17 obese men with a controlled weight-maintenance diet for 7 days. Subsequently, for 4 weeks each, these men consumed a high-protein and moderate-carbohydrate diet (HPMC; 139 g protein, 82 g fat, and 181 g carbohydrate/d) and a high-protein and low-carbohydrate (HPLC; 137 g protein, 143 g fat, and 22 g carbohydrate/d) diet, as part of a crossover experimental design. With the HPLC diet, the* Roseburia/Eubacterium rectale* group, an abundant Gram-positive family Lachnospiraceae, decreased (*P* = 0.001) as a proportion of total bacteria. While consuming the HPLC diet, the proportion of* Bacteroides *spp. decreased by 22% (*P* = 0.007), relative to the maintenance diet. In yet another study, utilizing a rat model of anorexia, researchers employed extreme food restriction plus increased activity; this combination of factors negatively impacted the quantity of health-promoting bacteria and enhanced the growth of bacteria which may be related to disruption of the gut mucosal barrier. Specifically, a significant increase was observed in the number of* Proteobacteria, Bacteroides, Clostridium, Enterococcus, Prevotella,* and* M. smithii* and a significant decrease in the quantities of Actinobacteria, Firmicutes, Bacteroidetes*, B. coccoides-E. rectale group, Lactobacillus, *and* Bifidobacterium* [19]. Further, excessive nutrient loading of the IM, or consuming a diet that eliminates one or more essential nutrients, may lead to an altered IM diversity because a small number of species overgrow and outcompete other flora (Figure 1). Consistent with this concept, decreased IM diversity has been linked with a diet that is high in fat and sugar, compared to a low-fat plant-based diet [20, 21].

Focusing on IM diversity, a study of elite Irish professional rugby players (*n* = 40; age, 29 ± 4 y; body mass index, 29 ± 3) evaluated the impact of exercise and dietary changes during preseason training. Investigators [22] performed a high-throughput DNA sequencing analysis of fecal microbiota with concurrent measurements of proinflammatory cytokines and metabolic health. Gut microbiota diversity was significantly greater in the athletes compared with size matched (body mass index > 28, *n* = 23) and age/gender matched (BMI ≤ 25, *n* = 23) control groups, with few differences seen between the two control cohorts. Athletes also had lower inflammatory responses and improved metabolic markers of health than the high BMI control group. Athletes consumed significantly more calories, protein, fat, and carbohydrate per day than both control groups. Microbiota diversity measures positively correlated with protein intake and plasma creatine kinase levels (a marker of extreme exercise), which suggested that both diet and exercise influenced the changes of microbial diversity [23]. Similarly, three investigations have shown that exercise training (20–66 min/d, 5 d/wk, 4–6 wk) alters the IM of rats [24], including pregnant and diabetic animals. Danish scientists at the University of Copenhagen [25] examined the impact of exercise training on the ability of exogenous i.v. lipopolysaccharide (LPS) to induce an inflammatory response (i.e., TNF-*α*, IL-6) in plasma and in biopsied skeletal muscle and adipose tissues of healthy young men. This research group reported that physical training status affected the ability to induce an acute inflammatory response, in a tissue-specific manner. In support of the above research, Campbell and Wisniewski [17] reviewed relevant publications and concluded that physical exercise training (a) is a potent intervention for the restoration of metabolic and gut health (i.e., subsequent to metabolic syndrome or gut inflammation), as well as for the diversification of the gut microbiota; (b) increases microbial diversity independent of diet; (c) increases antioxidant enzymes and anti-inflammatory cytokines; and (d) decreases proinflammatory cytokines [17]. These responses and adaptations explain, in part, why a program of regular physical exercise represents a cornerstone in the primary prevention of at least 35 chronic diseases [26].

## 4. The IM as a Predisposing Factor for EHS

The human IM is a community of commensal, symbiotic, and pathogenic microorganisms that reside within the human body in a complex bionetwork containing approximately 10^13^ cells. These microorganisms exceed the number of cells in the entire human body by a factor of 10 and their number of genes by a factor of 100. The IM may have a biomass as large as 2 kg, along the course of an 8 m intestine [27]. The total count of bacteria increases, moving from proximal to distal sites (Table 3): stomach, 0–10^3^; jejunum, 0–10^3^; ileum, 10^3^–10^7^; colon, 10^9^–10^12^; and feces, 10^10^–10^12^ [28]. Bacteria in the mammalian intestine produce and detect numerous extracellular signaling molecules, as one component in the multiple layers of communication between the IM, the host, and incoming pathogens [11]. For example, the gut IM constantly interacts with the host immune system and intestinal ultrastructure [29] (Table 4). Lipopolysaccharide (LPS), which originates from the outer membrane of Gram-negative bacteria, peptidoglycans, and bacterial DNA are recognized by receptors on luminal and intestinal immune cells [30]. Also, evidence demonstrates that commensal bacteria can communicate with incoming pathogens, control the expression of virulence, and inhibit the production of toxins [11]. Indeed, some of the signaling molecules which are sensed by incoming pathogens and which affect their function also may be produced by other pathogens.

**Figure 3:**
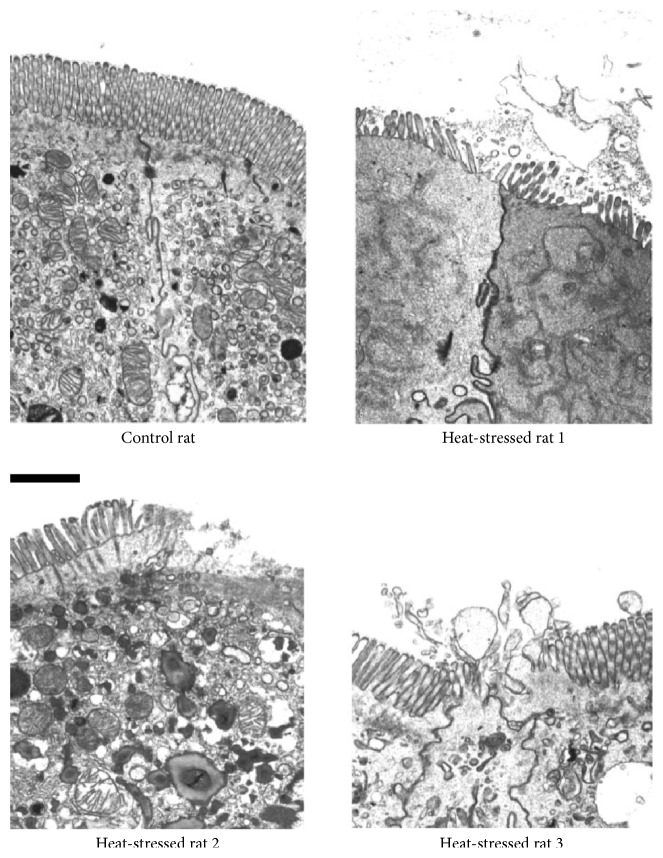
Electron micrographs of small intestine epithelial cells from control and heat-stressed rats. Damage to the microvilli and cell membranes is evident in the heat-stressed rats. Bar represents 1 *μ*m. Reprinted with permission from Lambert et al. [31].

The mucosal lining of the gastrointestinal tract protects the internal environment of the body from bacteria and endotoxins such as LPS [31]. This barrier consists of physical factors (i.e., enterocyte membranes and tight junctions), mucous secretion, and immune factors [31, 32]. Although a small amount of gastrointestinal permeability is normal, a healthy immune system prevents adverse effects; low levels of endotoxin are rapidly removed by monocytes, in particular the Kupffer cells that reside in liver tissues [33]. Dysfunction of or damage to the mucosal lining, and the adjoining single layer of epithelial cells, leads to increased permeability (i.e., diffusion of molecules from the lumen into blood) with mitochondrial swelling and vacuolization ([32]; Figure 3). Increased gastrointestinal permeability (i.e., barrier dysfunction) is of concern because it may allow passage of harmful substances (e.g., endotoxin, food antigens, digestive enzymes, and bile) from the intestinal lumen into blood. This can create local and/or systemic inflammation and endotoxemia. The latter is widely believed to be an etiological factor in EHS and its outcome [34].

The means by which LPS stimulates a proinflammatory immune response is well-described. The factors which have not yet been considered in the role of LPS-induced immune response and EHS pathophysiology are as follows: (1) the diversity of both immune responses to LPS and ligands that may stimulate the LPS responsive receptor on immune cells known as TLR-4 (toll-like receptor-4), (2) the diversity of LPS structural variants that can stimulate different immune responses, and (3) other endogenous molecules that can affect how LPS stimulates proinflammatory responses. Intriguingly, all three of these components have not yet been considered in published work on EHS pathophysiology.

**Figure 4:**
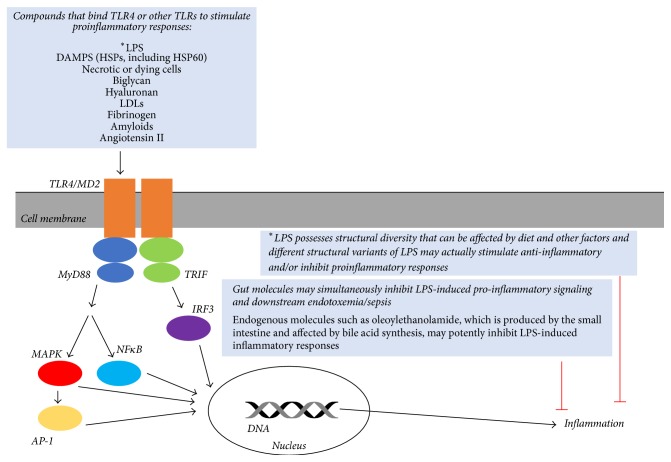
Many compounds other than lipopolysaccharide (LPS) can bind to a proinflammatory receptor on immune cells, TLR-4 (toll-like receptor-4). When TLR-4 and its accessory proteins, including lymphocyte antigen 96 (MD-2), are triggered by some of these compounds, two main pathways through initial signaling proteins MyD88 (myeloid differentiation primary response gene 88) and TRIF (TIR domain containing adaptor-inducing interferon-beta) stimulate a cascade of signaling to multiple proteins. The end result involves proteins that enter the nucleus, bind to DNA, and change gene expression to stimulate the production of proinflammatory proteins. Structural variations in LPS molecules can affect how robustly this response occurs, by affecting the strength of binding at TLR-4 to stimulate the initial signal. Additionally, other molecules or proteins may independently affect the strength of the proinflammatory response to LPS. MAPK (mitogen associated protein kinase); NF*κ*B (nuclear factor kappa light chain enhancer of activated B-cells); IRF3 (interferon regulatory factor 3); AP-1 (activator protein 1).

LPS is recognized by a cell membrane receptor named TLR-4 on immune cells (Figure 4). When TLR-4 is bound by LPS and other accessory proteins (e.g., lymphocyte antigen protein 96 termed MD-2) the complex stimulates intracellular communication by multiple pathways that converge when different proteins enter the nucleus and change gene expression, to stimulate production of proinflammatory proteins that may be released into circulation or signal to the immune system in other ways. Much of the work on endotoxemia and sepsis in EHS pathophysiology has focused on LPS. However, there are multiple ligands (i.e., molecules that can bind to TLR-4) in addition to LPS that can stimulate TLR-4 to produce the same or an augmented proinflammatory response. A 2010 review [35] described over 20 endogenous or naturally occurring (versus synthetic or pharmacological) TLR-4 ligands that are capable of stimulating proinflammatory processes; this review emphasized concerns regarding contamination of experimental systems by these ligands. Thus, the mechanistic diversity of TLR-4 stimulation should be considered in hypotheses regarding endotoxemia/sepsis and EHS. For example, heat shock protein 60 (HSP60), a protein that increases with exercise [36], is also considered to be, in its protective role, a DAMP (i.e., danger-associated molecular pattern). In this role, HSP60 is able to bind to receptors like TLR-4 on immune cells to stimulate an immune response. It is likely that signals from LPS, DAMPs like HSP60, and other TLR-4 ligands can concurrently magnify the proinflammatory response to exercise-heat stress and associated tissue damage during EHS.

Another consideration that can affect how LPS may play a role in EHS is the diversity of the LPS molecule itself. LPS is made of 3 domains including a core of oligosaccharides bound to molecules termed the Lipid A anchor and the O antigen. Lipid A is known to be the primary component of LPS that stimulates the immune response resulting in endotoxemia and sepsis. Both the degree of molecular modifications of Lipid A and genetic differences in the TLR-4 protein receptor among individuals can have a notable impact on LPS responsiveness and the strength of a proinflammatory response [37]. Variations in Lipid A that arise from different bacterial species can also affect how TLR-4 recognizes LPS and how potently it stimulates a proinflammatory response [38]. These potential variations in Lipid A and TLR-4 structure have not yet been studied in the hypothetical role of the IM in EHS pathophysiology.

A third major consideration involves the variation of ligands that directly bind TLR-4 and the diversity of molecules that can independently affect TLR-4 stimulation of an immune response; some of these can be produced in and leaked from the gut. One highly intriguing example is that of oleoylethanolamide (OEA), a small molecule produced in the small intestine. Experiments have demonstrated that OEA can directly inhibit LPS-induced TLR-4 stimulation of the immune response by defined mechanisms [39]. Treatment of human cells with OEA has prevented full gene expression associated with LPS-induced stimulation of TLR-4 and has significantly reduced proinflammatory signaling. Thus, it is evident that there may be multiple inputs to regulate the immune responses which occur during exercise-heat stress, to either promote or inhibit the progression of EHS.

Table 4 describes influences which the IM may have on immune function and inflammation [40]. These effects include degradation of toxins produced by pathogenic bacteria, modulation of antibody production in response to antigens, production of metabolites which have proinflammatory and anti-inflammatory actions, and release of LPS from the wall of Gram-negative bacteria when they die. Importantly, Table 5 notes that LPS in plasma is a potent agonist for the release of proinflammatory cytokines during EHS. In severe EHS cases, multiple-organ dysfunction or failure occurs, mediated by leakage of LPS into the circulation [41–43]; this may culminate in systemic hypotension and cardiovascular shock [44–47]. In dogs, intravenous injection of* Escherichia coli* endotoxin is characterized by an immediate, rapid decline of blood pressure with a simultaneous elevation of hepatic portal vein pressure, signifying trapping of blood in the liver [48]. The combined effects of LPS and hyperthermia also stimulate blood coagulation. Autopsy findings typically include hemorrhage and microthromboses in the splanchnic organs, (i.e., intestine, liver, lungs, kidneys, pancreas, and spleen), heart, brain, cornea, and skin [43, 49–51].

## 5. Factors Which Increase Intestinal Permeability and Plasma LPS

**Figure 5:**
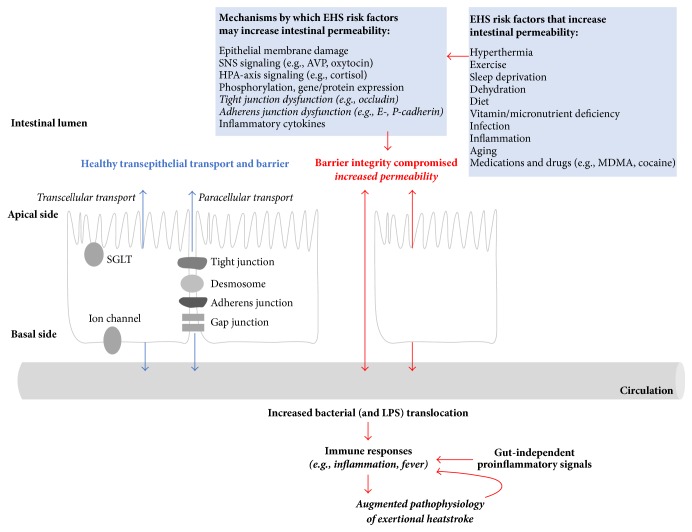
An intact intestinal epithelium allows efficient transepithelial transport across cells (transcellular transport) and between cells (paracellular transport) via (1) channels directly embedded in the membrane such as SGLT (sodium-dependent glucose transporter), ion channels, and aquaporins and (2) control of gap junctions, adherens junctions, desmosomes, and tight junctions between epithelial cells. EHS risk factors including hyperthermia, exercise, dehydration, sleep deprivation, drugs, and diet are known to increase intestinal permeability via multiple independent mechanisms. When intestinal barrier integrity is compromised, increased permeability results in bacterial or endotoxin translocation into circulation. Bacterial components may stimulate an immune response that results in signaling that promotes positive feedback of inflammation and inflammation-associated symptoms such as fever. Simultaneously, stress or tissue injury also may signal to enhance proinflammatory immune responses via gut-independent signals such as DAMPs (danger-associated molecular patterns) including heat shock protein 60, which are increased during cell/tissue stress. SNS, sympathetic nervous system; AVP, arginine vasopressin; HPA, hypothalamic-pituitary-adrenal.

Enterocytes are the predominant epithelial cells of the gut mucosal lining and form a physical barrier that limits passage of small molecules (molecular mass ≤ 500 Daltons) [52] (Figure 5). Enterocyte cell membranes are folded into finger-like microvilli which increase the surface area available for digestion and absorption of electrolytes, water, and other nutrients. Permeability can be increased via the following: (1) transcellular or paracellular active transport and endocytosis using channels (e.g., sodium-dependent glucose cotransporters (SGLTs), aquaporins), (2) changes in gap junctions, and (3) changes in adhesive complexes (junctions). Adhesive junctions include the following: (1) plasma membrane structures called tight junctions (TJ) comprised of multiple proteins (e.g., claudins, occludins, junctional adhesion molecules (JAMs)); zonula occludens (ZO proteins, actin); (2) multiprotein subjacent adherens junctions (AJ) including cadherin and catenin proteins; and (3) desmosomes.

Multiple risk factors for exertional heatstroke, including hyperthermia, sleep deprivation, dehydration, and aging, have been shown to directly affect the integrity of the intestinal barrier and thus intestinal permeability (Figure 5). Mechanisms by which transcellular and paracellular transport are affected to impact gut permeability include direct damage to tissue [31], sympathetic nervous system signaling [53], hypothalamic-pituitary axis activity [54, 55], phosphorylation [56], gene/protein expression [57], and inflammation-induced damage and signaling. It is intriguing to consider the multiple independent and perhaps cumulative effects of different stressors associated with EHS on intestinal permeability and downstream proinflammatory signaling.

### 5.1. Hyperthermia

Severe hyperthermia can disrupt the membrane structure of enterocytes (Figure 3), allowing endotoxin (i.e., LPS) to pass into the circulation, stimulating a systemic immune response characterized by proinflammatory cytokine release [58]. This response likely involves tight junctions, which exhibit increased leakiness to bacterial LPS (molecular mass 10–20,000 Daltons) after exposure to temperatures as low as 38.3°C, with larger molecules penetrating at temperatures higher than 41.5°C [59, 60]. The release of LPS from Gram-negative bacteria embedded in the mucosal lining, and its appearance in blood, is a potent agonist for the release of cytokines (e.g., interleukin-6, IL-6; tumor necrosis factor *α*, TNF-*α*), which exacerbate systemic inflammation in cases of severe heatstroke [42]. Case reports of EHS (i.e., considered to be a medical emergency) have involved the proinflammatory cytokines IL-6, TNF-*α*, and IL-1, as well as the anti-inflammatory cytokines IL-1RA, IL-10, and soluble TNF receptors [61].

Gaithram and colleagues [62] studied the role of LPS in EHS pathophysiology by passively heating 11 anesthetized monkeys to a rectal temperature of 43.5°C, then allowing them to cool in a 25°C environment; five received a prophylactic intravenous (i.v.) dose of hyperimmune plasma (i.e., containing antibodies to counteract LPS), and six control animals received an equivalent dose of nonimmune plasma. The former group experienced little or no increase of plasma LPS and 100% survival; 83% of the control group died, with elevated plasma LPS concentrations. This research team [63] also investigated the prophylactic administration of a nonabsorbable antibiotic via nasogastric tube (kanamycin, twice daily for 5 d), prior to passive heating (44.5°C rectal temperature). No increase of plasma LPS was observed in the four antibiotic-treated animals. In control animals, receiving no antibiotic, plasma LPS increased from 0.04 (37.5°C) to 0.06 (39.5°C) and 0.31 ng·ml^−1^ (44.5°C). These data suggested that the increased plasma LPS during heat stress originated mainly from the gut and supported research involving anesthetized dogs. Bynum et al. [64] reduced the intestinal stool and bacterial contents of dogs by administering antibiotics, cathartics, and enemas, before heating to a rectal temperature of 43.5°C. Following the reduction of gut flora, the incidence of 18 h survival rose from 20.0% to 70.6%. Antibiotics administered after heatstroke resulted in similar survival rates in experimental and control groups. Subsequently, Bouchama and colleagues [42] were able to describe, in baboons, differences between mild-to-moderate EHS and more severe cases. Nonsurvivors displayed significantly greater inflammatory activity and tissue injury than survivors. The animals with severe heatstroke exhibited a marked increase in plasma IL-6 levels which was strikingly similar to humans with near-fatal heatstroke, in whom the highest plasma IL-6 levels correlate with poor outcome [44]. Studying cytokine responses, Tracey et al. [65] passively immunized anesthetized baboons with TNF-*α* antibody fragments and then administered an i.v. LD_100_ dose of live* Escherichia coli* at two subsequent time points. Administration of antibodies 2 h before bacterial infusion provided complete protection against shock, vital organ dysfunction, persistent stress hormone release, and death. However, when antibodies were administered only 1 h before the bacterial challenge, critical organ failure occurred with no shock. Control, nonimmunized animals experienced hypotension followed by lethal renal and pulmonary failure. These experiments indicated that TNF-*α* mediated fatal bacterial endotoxic shock.

Based on the animal studies described above, increased plasma LPS is suspected to be an important etiological factor in the circulatory shock that accompanies advanced EHS in humans [31, 43, 66]. For example, elevated plasma LPS concentration reduces stroke volume, heart rate, and cardiac output by 30–50% in mice [67]. Although few studies have cultured bacteria from heat stroke patients, increased plasma LPS has been observed in (a) an EHS patient who presented with body temperature > 43°C and severe liver necrosis associated with Gram-negative sepsis [68] and (b) nonexertional heatstroke patients with core temperatures of ~42°C [69]. Under normal circumstances, endotoxin that leaks through the intestinal wall into the circulation is rapidly detoxified and inactivated in the liver [33]. However, under severe heat stress, the reduction in hepatic portal vein blood flow combined with thermally altered hepatocyte function severely reduces the capacity to detoxify a surge of endotoxin [48, 70]. The resulting increase of circulating LPS levels may result in fever, shivering, dizziness, nausea, vomiting, and diarrhea [71].  Table 6 presents the signs, symptoms, and laboratory variables which may be observed in cases of EHS; this table also illustrates multiple-organ dysfunction or failure. The majority of the factors in Table 6 also may be observed in patients who experience sepsis [43], a life-threatening condition that arises when the body's responses to an infection injure its own tissues and organs [47]. Sepsis and septic shock are described in greater detail below, in the section titled Sleep Deprivation.

### 5.2. Intense or Prolonged Exercise

Planned moderate-intensity exercise programs across several weeks represent a cornerstone in the primary prevention of chronic diseases [26] but stand in stark contrast to the acute physiological responses which occur during high-intensity exercise. For example, it is widely recognized that high-intensity exercise in a mild environment causes intramuscular pH to fall from 6.9 at rest to 6.4, and plasma pH to decrease similarly, after both intermittent and continuous maximal exercise [72]. But, to our knowledge, only one study has reported the pH of intestinal mucosa following maximal exercise in a mild environment [73]. The experimental protocol caused intramucosal pH to fall from 7.3 to 6.8, at the end of 30 minutes of maximal rowing ergometry. The authors attributed this acidotic state to decreased blood flow and intestinal hypoxia; other authorities have theoretically implicated [32, 34, 74] and empirically measured [75] energy (i.e., ATP) depletion in this process (a complete description of a theoretical energy depletion model involving EHS appears in [76]). Subsequently, Menconi and colleagues [77] reported that acidosis induced hyperpermeability in laboratory-cultured epithelial cells; a medium maintained at pH 5.4 increased both transcellular and paracellular movements of macromolecules. Thus, both high-intensity exercise and severe EHS induce lactacidemia and metabolic acidosis [43, 51]. Indeed, the plasma lactic acid concentrations of EHS patients reflect whole-body metabolic acidosis [78] and are significantly correlated with neurologic morbidity and mortality [79, 80]. Although it is unlikely that either of these two studies [72, 73] induced a markedly elevated body temperature, severe hyperthermia of internal organs is a primary, noxious etiological factor that reduces splanchnic blood flow [81]; increases intestinal permeability [31]; injures intestinal epithelial cells [34]; and stimulates hypercoagulability. Thus, maximal exercise and a rectal temperature > 41°C induce similar detrimental effects (i.e., resulting in endotoxemia) which likely are additive, when they occur simultaneously, as in EHS [82]. Experiments involving nonhuman primates suggest that LPS becomes increasingly important as a pathological agent as internal body temperature approaches 43.5°C [43]; above this temperature, direct thermal damage to enterocytes (Figure 3) and brain tissue is probably a more significant noxious stimulus [62]. Further it is possible, but undocumented, that cells of the intestinal epithelium generate lactic acid locally, via anaerobic metabolism, when splanchnic blood flow is low and splanchnic tissue is hypoxic [75, 77]. This is hypothetically significant because lactic acid penetrates the cytoplasmic membrane of Gram-negative bacteria and liberates LPS [83], more effectively than hydrochloric acid and EDTA—a preservative that prevents bacterial food spoilage.

Prolonged exercise also may cause plasma LPS to increase independently, or in combination with hyperthermia. For example, after a long distance triathlon (3.8 km swimming, 185 km cycling, and 42.2 km running) involving high altitude (3,200 m) and a maximal air temperature of 32.1°C, mild endotoxemia (68% of athletes) was observed [84]. Similarly, following a marathon footrace, eight out of 18 runners exhibited very mild endotoxemia (5 to 14 pg·ml^−1^), whereas one athlete had a high LPS level of 72 pg·ml^−1^ [85]. In mild ambient conditions (20–23°C), increased plasma LPS was observed in 81% of ultraendurance athletes who had completed an 89.4 km footrace [86] and in healthy runners following 1 h of strenuous treadmill exercise (80% of maximal aerobic power; final rectal temperature, 39.6°C) but not at two slower running speeds with lower rectal temperatures [87]. This suggests that the degree of LPS translocation into blood is proportional to the duration and intensity of exercise, during training [88] and competition [17]. The mechanisms of this increased gastrointestinal permeability theoretically involve reduced splanchnic blood flow which occurs during exercise [89, 90], the resultant hypoxic or acidic intestinal tissue, and/or reduced convective heat loss via blood, from central organs to the skin [31]. Intestinal ischemia has been shown to occur within 10–20 min of high-intensity exercise [91].

An interesting theory has emerged regarding the effects of repeated exercise-heat stress [92]. This paradigm involves the routine, repeated stimulation of host immune defenses during exercise-heat stress, via translocation of small amounts of LPS into the circulation of healthy individuals [93]. This theory applies to the chronic state known as “heat hardening” (i.e., the improved heat tolerance exhibited by long-term residents of hot climates) and to the superior but transient heat tolerance of highly trained individuals, gained by their daily exercise in the heat [94]. As noted in the previous paragraph, evidence shows that endurance athletes have elevated LPS antibody (anti-LPS IgG) concentrations at rest [84, 85, 88]. This suggests that individuals participating in regular strenuous physical activity or endurance training may develop an improved endotoxin tolerance [95] due to small, repeated exposures to LPS [94, 96, 97], in a form of self-immunization [88].

Another intriguing theory regarding the IM recently resulted from laboratory experiments which evaluated the endurance exercise performance of mice. Hsu and colleagues [98] examined whether gut bacteria would alter antioxidant enzyme levels and the exercise performance of 12-week-old male mice. Three varieties of animals were examined: mice containing no microorganisms, including the intestine (GF, *n* = 8); those that were free of a specific pathogen by routine testing (SPF, *n* = 8); and germ-free mice intentionally inoculated with* Bacteroides fragilis* bacteria (BF, *n* = 8).* Bacteroides fragilis* are a Gram-negative anaerobic bacteria (Table 2) which provides beneficial effects to humans and animals. The absence of bacteria (GF group) decreased exercise performance time, but inoculation of germ-free mice with* Bacteroides fragilis* prevented the decline of endurance exercise time. In stating their unique findings, the authors noted that different microbial status altered exercise performance, possibly by altering innate antioxidant systems, as evidenced by lower levels of glutathione peroxidase and catalase in the serum and liver tissue of GF mice. Both of these enzymes normally defend against oxidative damage but become weaker during chronic fatigue and intense exercise [99]. Theoretically, improved activities of these enzymes (i.e., due to a consistent, progressive exercise training program) may counteract fatigue [100]. To our knowledge, this is the first evidence that the IM may influence endurance exercise performance directly. We find these results to be meaningful, considering that the IM theoretically may influence exercise performance in other ways, including carbohydrate and lipid absorption by enterocytes, energy metabolism, insulin sensitivity, free fatty acid oxidation, and electrolyte-water absorption [101–103]. Further research is required to confirm these findings.

### 5.3. Exercise-Heat Acclimatization

During 10–14 days of exercise or labor in a hot environment, adaptations occur which improve exercise-heat tolerance, reduce physiological strain, and reduce the risk of EHS. Involving critical organ systems, these adaptations include expanded plasma volume and total body water, increased sweat rate, reduced heart rate, deep body temperature, sodium concentration in sweat and urine, and perceived exertion during exercise [104]. This process is named heat acclimatization in a natural environment and heat acclimation when exercise-heat exposure occurs in a controlled environmental chamber. Two studies are relevant to the influence of exercise training and heat acclimation on increased intestinal permeability. First, a research team led by Amorim et al. [105] supervised a 10 d heat acclimation protocol (42°C ambient temperature; walking or running for 100 min·d^−1^; 9 healthy adults). They assayed the plasma proinflammatory and anti-inflammatory cytokines which LPS stimulates (TNF-*α*, IL-1*β*, IL-6, and IL-10), heat shock protein 72 (Hsp72), and the enzyme lactate dehydrogenase as a marker of cell damage, before and after the 10 d intervention. This heat acclimation protocol did not alter the release of cytokines, nor did it change the degree of cellular damage. The level of Hsp72 increased across 10 d, in contrast with a study conducted in our laboratory, in which heat shock protein 70 was unaffected by 11 d of similar exercise-heat acclimation [106]. The authors [105] concluded that a heat acclimation program, which limits internal body temperature to <39°C, may not provide cellular tolerance to the stressors encountered during EHS. Second, Lim and colleagues [96] observed 18 trained distance runners during 14 d of endurance exercise in a mild environment. Before (T1) and after (T2) the training intervention, these men undertook a heat stress test (70% of their maximal aerobic power until rectal temperature reached 39.5°C) in a hot 35°C environment. Plasma LPS levels, and the cytokines IL-6 and TNF-*α*, were measured. The heat stress test induced mild endotoxemia during T1 and T2 but was well tolerated. The 14-day training program reduced plasma LPS concentrations, before exercise and at 1.5 hours after exercise. The authors concluded that these adaptations were not likely due to improved aerobic fitness (i.e., because the runners began this study in a highly trained state) or heat acclimation (i.e., because the only heat exposures were the exercise-heat stress tests). Rather, the authors proposed that frequent exposure to sublethal doses of LPS (i.e., as occurred between T1 and T2, during daily workouts in a mild environment) may inhibit LPS translocation and enhance LPS clearance by the liver [96]. Given the small number of publications regarding intestinal permeability during planned exercise-heat acclimatization, additional research is required.

### 5.4. Sleep Deprivation

Strenuous exercise can increase the plasma cytokine concentrations of IL-1*β*, TNF-*α*, and IL-6 in physically trained adults [107, 108]; these increases may persist for hours to days. Interestingly, both IL-1*β* and TNF-*α* are involved in the regulation of sleep; they are somnogenic, in addition to their role in inflammation [43]. Other cytokines also may influence sleep: IL-1 is produced as a result of sleep deprivation [109], and a stress-induced plasma IL-10 increase may alter sleep pattern and times [110]. These findings support earlier research studies that identified strong associative relationships between changes in sleep duration and immune function [111–113]. Further, sleep loss was the most common (i.e., out of 16 total reported) predisposing factor or personal warning signal for EHS, experienced by 70% of former EHS patients. Thus, even though sleep deprivation is considered to be a risk factor for EHS, up to 64 h of sustained wakefulness in young, healthy individuals produced only minor, nonspecific clinical signs [114]; similarly, 9 days of voluntary sleep deprivation (i.e., with no inner organ hyperthermia) ended in collapse and hospitalization due to unspecified causes [115]. Thus, specific health impairments have not been definitively linked to sleep deprivation, the mechanism(s) are unknown, and no descriptive clinical signs have been identified for human sleep deprivation [116].

Observations of sleep-deprived animals suggest that host immune defense failure is central to the adverse effects of sleep deprivation, appearing as bloodstream infection. Specifically, bacterial and LPS translocation, and their pathogenic sequelae, provide possible mechanisms by which sleep deprivation appears to adversely affect health [116]. In severely sleep-deprived rats, for example, the proximal cause of death was infection by anaerobic bacteria from the IM, such as* Pseudomonas aeruginosa, Klebsiella pneumoniae, Staphylococcus aureus, Streptococcus agalactiae, *and* Corynebacterium jejeikum* [117]. These microorganisms do not cause primary bacteremia or threaten life unless the host is immunocompromised, but their presence in blood is highly lethal in humans [116]. Without the experimental administration of any agent except sleep loss, prolonged wakefulness produced a life-threatening hypermetabolic and systemic inflammatory state in rats that was not accompanied by the usual diagnostic symptoms of fever and large tissue inflammation. Because of the nonlocalized and toxic-like nature of sleep deprivation (i.e., the rapid reversibility of debilitation via sleep), without evidence of permanent damage, the most plausible cause of death [117, 118] from sustained sleep deprivation was septicemia (i.e., bloodborne bacterial infection).

More than twenty years after these animal experiments (i.e., despite numerous publications regarding the pathobiology, biochemistry, immunology, management, and epidemiology of sepsis), a 19-member task force [47] acknowledged that sepsis, septic shock, and systemic inflammatory response syndrome (SIRS) are difficult to diagnose. This task force also concluded that (a) sepsis is not a specific illness but a syndrome that encompasses an uncertain pathobiology and is without a validated standard diagnostic test; (b) septic shock is a subset of sepsis in which the underlying circulatory and cellular/metabolic abnormalities increase mortality substantially; and (c) the term* severe sepsis* is redundant. Although SIRS has been implicated in the pathophysiology of EHS [82], and although the clinical and laboratory results observed in cases of EHS (Table 6) are similar to sepsis and septic shock [43, 44, 119, 120], the relationship remains theoretical. Importantly, both EHS and septic shock exhibit systemic inflammation leading to a syndrome of diffuse, nonlocalized multiorgan dysfunction or failure (Table 6; [44, 47]). Indeed, EHS has been named “heat sepsis” because of the essential etiological role that LPS plays [121].

### 5.5. Dehydration

Dehydration may contribute to reduced intestinal blood flow, causing tissue hypoxia and heightened intestinal permeability [122]. Also, human prehydration with intravenous fluid (1.5 L of 2.5% glucose/0.45% NaCl), 1 h prior to an injection of* Escherichia coli *purified LPS endotoxin, may shift the resulting cytokine balance towards a more anti-inflammatory pattern, with a reduction of symptoms [123] and an outcome that is similar to the successful treatment of sepsis (i.e., life-threatening organ dysfunction caused by a dysregulated host response to infection [47] via i.v. fluid [124, 125]). To evaluate the effects of dehydration on gastrointestinal permeability, Lambert and colleagues [122] conducted a laboratory study in which twenty trained endurance athletes (11 men, 9 women) ran three times, in a 24°C mild environment, for 60 min at 70% of maximal aerobic power. During three double-blinded, randomized experiments, participants consumed either a 4% glucose solution (GLU), a placebo fluid (PLA), or no fluid (NF). Running increased GI permeability compared to rest, with the greatest permeability occurring during the NF trial. GI permeability did not significantly increase above resting levels when fluid was ingested at a rate sufficient to offset sweat losses (GLU and PLA). Although the increase in GI permeability in the NF trial did not result in greater gastrointestinal symptoms or cause any other noticeable problems, the authors [122] commented that barrier dysfunction likely would have worsened, with more prolonged or intense exercise.

### 5.6. Diet

Commensal gut microbes digest food components and release a variety of metabolites that are involved in the mutualistic balance achieved between microbiota and the host [126] (i.e., both partners benefit). The IM and its collective genomes (i.e., the microbiome) accomplish important metabolic functions and provide us with the ability to harvest otherwise inaccessible nutrients and energy. For example,* Bacteroides thetaiotaomicron* is prominent in the distal intestine of adult humans. This successful commensal anaerobe has an exceptional capacity for digesting otherwise indigestible dietary polysaccharides. Its glycobiome contains one of the largest sequenced gene ensembles that metabolizes carbohydrates (i.e., 226 predicted glycoside hydrolases and 15 polysaccharide lyases) [127]. By contrast, the human genome contains only 98 known or presumed glycoside hydrolases and is deficient in the enzyme activities required for degradation of xylan-, pectin-, and arabinose-containing polysaccharides that are common components of dietary fiber [128]. Numerous other anaerobic bacteria are capable of digesting plant materials, including nondigestible carbohydrates, nonstarch polysaccharides, resistant starch, and oligosaccharides [129] which humans cannot process. The metabolic intermediates and end-products which result from bacterial fermentation of these materials (Table 2) include short-chain fatty acids (SCFA; butyrate, propionate, acetate, and succinate), modified bile acids, and vitamins [130–132]. The SCFA, especially butyrate and propionate, regulate host glucose metabolism and immunity favorably, resulting in reduced proinflammatory cytokine production [130, 133]; are important in water and electrolyte absorption in the colon; modulate colon and liver blood flow; encourage colon mucosal integrity [102]; reduce intestinal permeability; and modulate chronically elevated plasma LPS [134].

Tables 3–5 describe a few of the many interactions between diet (i.e., nutrients, biochemical substrates) and physiological functions (e.g., digestion) that are relevant to EHS. For example, during daily activities, diet modulates inflammation and immune function, pH of the mucosal lining surrounding intestinal epithelial cells, intestinal permeability, and glucose homeostasis, insulin secretion, and energy metabolism [103, 133, 135]. In our opinion, these effects qualify diet as a predisposing factor for EHS.

In recent years, research involving the interactions between diet, the IM, and immune functions have focused on obesity, insulin resistance, and the metabolic syndrome. A large body of research in mice indicates that the IM is involved in the development of these disorders and that chronic, low-grade inflammation is part of their etiologies. This condition has been named metabolic endotoxemia because elevated plasma LPS levels have been identified as the prime factor in the low-grade inflammation and insulin resistance which occurs in the liver, muscles, and adipose tissue [40]. Metabolic endotoxemia is an example of dysbiosis (i.e., a disruption of the normal, healthy balance between the gut microbiota and host) that reflects lifestyle decisions (e.g., diet, exercise) and host genomic interactions [17]. Conducting an exquisitely designed series of mice experiments, Cani and colleagues reported the following findings. First, altering the bacterial composition of the IM in two strains of mice (i.e., nutritionally and genetically obese) increased plasma LPS and low-grade inflammation, type 2 diabetes, and insulin resistance [136, 137]. Second, the relevance of LPS signaling to the development of diet-induced low-grade inflammation was verified in mice lacking the immune cell receptor for LPS (toll-like receptor-4, TLR-4). Third, they demonstrated that metabolic endotoxemia, produced by chronic subcutaneous infusion of LPS (i.e., mimicking metabolic endotoxemia), significantly induced insulin resistance and inflammation [40]. Fourth, researchers interfered with LPS signaling by administering subcutaneous LPS quenchers (i.e., antibiotic or endotoxin inhibitors) for 4 weeks in genetically obese mice; significant decreases of inflammation occurred, together with improved glucose tolerance and insulin resistance [136]. Other experiments, involving genetically altered strains of mice [138] and antibiotic treatment [139], similarly demonstrated the contribution of gut-derived LPS to metabolic endotoxemia. This inflammatory abnormality is only one of a large variety of human diseases that are classified as immune system disorders and which involve increased plasma LPS (Table 5).

The relationships between a high-fat diet, LPS, obesity, and Type 2 diabetes also have been investigated in human test subjects. For example, Erridge et al. [35] and Ghanim et al. [140] examined baseline endotoxin concentrations in healthy human subjects and found that a high-fat meal (versus no meal) or a high-fat, high-carbohydrate meal (versus a high-fiber and fruit meal) increased plasma LPS concentration. Dogan and colleagues [141] reported a similar effect after consuming a high fructose diet. A link between metabolic endotoxemia, high energy intake, and a high-fat diet also was observed in a cohort of 201 men [142] and in multiple independent studies [143–145]. One of these [146] reported that LPS can increase adipose proinflammatory cytokine release (e.g., TNF-*α* and IL-6) and insulin resistance. Considered together, these mice and human investigations strongly suggest that the IM and diet contribute to LPS-related metabolic endotoxemia independently and concurrently [40].

Relevant to EHS, we propose that a state of chronic metabolic endotoxemia [17, 40, 136, 137], involving increased intestinal permeability, can be potentiated or multiplied by hyperthermia, high intensity/prolonged exercise, reduced blood flow to splanchnic organs, enterocyte hypoxia, and acidity, as described above. An animal model of heatstroke supports this proposition. Lin et al. [147] observed rats with a preexisting inflammatory state, induced by exogenous administration of i.v. LPS, at the point that rectal temperature reached 42.5°C. When compared to a saline control group, the LPS-treated rats exhibited greater inflammation (i.e., increased plasma IL-6, TNF-*α*, and IL-1*β*), hypercoagulation, and multiorgan dysfunction (i.e., increased plasma creatinine, blood urea nitrogen, alkaline phosphatase, aspartate aminotransferase, and alanine aminotransferase). The authors concluded that a preexisting inflammatory state can exacerbate the multiorgan injury during heat exposure.

### 5.7. Chronic Inflammation and Illness

LPS affects the brain and central nervous system in several ways. Fever, for example, is a regulated rise of body temperature, provoked by invading microorganisms and LPS. A widely accepted theory states that LPS stimulates bloodborne immune cells (i.e., monocytes and macrophages) to produce cytokines (e.g., IL-1*β*, IL-6, IL-8, TNF-*α*, and interferon-*γ*) which, in turn, mediate fever by stimulating prostaglandin E_2_ in the organum vasculosum laminae terminalis (OVLT) of the brain; OVLT nerve impulses then stimulate the hypothalamus, which regulates whole-body heat balance. An alternative theory suggests that LPS influences the OVLT-PAH area of the brain via peripheral vagal nerves which sense the presence of LPS in the liver [148]. Regardless of the pathway, much evidence indicates that immune system responses to LPS have acute central effects [149]. In cases of chronic inflammation, lymphocytes and macrophages predominate, and the cytokine IL-6 is critical to the control of local and system-wide inflammation [150] and the transition from acute to chronic inflammation. In chronic autoimmune diseases (i.e., rheumatoid arthritis, lupus, and psoriasis), plasma IL-6 is elevated and sustains inflammation via T-cells and B-cells [151].

Crohn's disease (CD) and ulcerative colitis (UC) are the two primary chronic inflammatory bowel diseases (IBD). Several lines of research evidence suggest that the IM influences the pathogenesis of these diseases. Although most studies of IM influences have analyzed fecal contents, the work of Frank and colleagues [152] analyzed tissues obtained from CD patients, UC patients, and non-IBD control subjects during surgery at a variety of small intestinal sites; tissues included both pathologically normal and abnormal tissues. Their comprehensive rRNA sequence analysis of the IM indicated that a subset of CD and UC samples were characterized by depletion of commensal bacteria, notably members of the phyla Firmicutes and Bacteroidetes. Further, patients stratified on the basis of their IM (a) showed that CD represents a spectrum of disease states, and (b) suggested that treatment of some forms of IBD may be facilitated by rectifying microbiological imbalances [152]. Chronic, low-grade systemic inflammation induced by LPS has been associated with other diseases and lifestyle factors, including the following: insulin resistance, obesity, and diabetes [153], colorectal cancer [154], and alcoholism and liver cirrhosis [155, 156]. Regarding the latter conditions, (a) selective intestinal decontamination with antibiotics results in a decline of plasma LPS and attenuated liver damage in animal models of alcoholic liver disease [157] and (b) dysbiosis of the IM occurs in alcoholics, who have a lower abundance of butyrate-producing bacteria (i.e., generally believed to be anti-inflammatory) and a higher abundance of bacterial phyla believed to be proinflammatory [156, 158].

### 5.8. Acute Illness and Infection

Individuals with a preexisting barrier dysfunction, bacterial infection, or febrile illness may be at greater risk of developing EHS. When these conditions exist prior to exercise, internal body temperature may begin well above 37°C, and it may reach the 40°C clinical threshold of EHS sooner than in healthy individuals [3, 159]. Evidence for this statement exists in a published case report of a 21-year-old man who exhibited slight hyperthermia (39.1°C) during the third consecutive day of laboratory-controlled, light treadmill exercise (100 min, 40°C environment). His elevated rectal temperature, according to the authors, was associated with an acute local infection of a foot friction blister [160], diagnosed as cellulitis and treated with antibiotics. Because the incubation period for bacterial cellulitis extends across several days, this infection was subclinical (i.e., not recognized by the young man) before abnormal thermoregulation occurred.

Not surprisingly, a number of studies have implicated illness with fever as a predisposing factor to EHS [50]. One case report involved a 19-year-old man who experienced EHS twice during prolonged marches, separated by only one month [161]. His first EHS involved clinically overt gastroenteritis, which diminished his exercise-heat tolerance. The authors suggested that a subclinical infection may have predisposed the second event. Additionally, twelve years of epidemiological data, recorded in a military hospital, showed that more than 95% of EHS patients had mild fever, upper respiratory tract infection, or diarrhea prior to their event [162]. However, if a highly motivated young individual does not perceive overt EHS symptoms, or has mild symptoms of EHS, he/she may exercise to the point of collapse when under the discipline of sport competition, work, or military maneuvers. Under other circumstances, these same individuals typically would have remained at home when ill and would have rested when fatigued [51].

Healthy young individuals who experience EHS are generally unaware of the subtle signs or seriousness of their increasing hyperthermia during exercise (i.e., rectal temperature approaching 40°C), relative to the familiar signs of fatigue or exhaustion [74, 163]. Premonitory signs of illness, during the days prior to EHS, may be their only advanced warning. Such signs were reported by 10 infantry soldiers (mean ± SD; age, 26 ± 2 y; body fat, 15 ± 2%; maximal oxygen consumption, 50 ± 2 ml·kg^−1^·min^−1^) who participated in prospective laboratory testing, 61 ± 7 days after an episode of EHS [164]. Six of these former patients recognized prodromal signs of impending illness (e.g., headache, dizziness, lack of coordination, and disorientation; Table 6) during the 5 d prior to EHS. Interestingly, 80% of these men experienced EHS (rectal temperature, 41.0 ± 0.2°C) while running at a relatively slow pace (12.1–13.8 km·h^−1^), during morning (0630–1000 h) group physical training (distance, 6.1 ± 1.6 km), when the environmental temperature was not harsh (dry bulb temperature, 23 ± 3°C) but the relative humidity was high (88 ± 11%). This implicated a preexisting illness as a predisposing factor. Gastroenteritis (i.e., stomach or intestinal flu) is a prime example, involving symptoms such as watery diarrhea and vomiting (i.e., resulting in dehydration), abdominal pain, cramping, fever, nausea, and headache. Viral pathogens in the genera* rotavirus* and* norovirus*, as well as bacterial pathogens such as* Escherichia coli* and* Salmonella enterica*, are the most common cause of gastroenteritis.

Lin and colleagues [147] investigated the effects of a preexisting LPS-induced acute inflammatory state on organ function. They compared normothermic rats treated with normal saline (CON), normothermic rats treated with LPS (L), heat-stressed rats treated with normal saline (H), and heat-stressed rats treated with LPS (HL). The H and HL groups were exposed to a 43°C environment for 53 minutes. Measured variables included proinflammatory cytokines (IL-1*α*, IL-6, and TNF-*α*), biomarkers of organ dysfunction (liver ALT and AST, renal creatinine, and BUN), and blood coagulation factors (prothrombin time, D-dimer, and protein C). These measured variables increased progressively, as follows: CON > L > H > HL. This supported the concept that preexisting endotoxemia exacerbated overproduction of proinflammatory cytokines, hypercoagulation, and organ injury [147]. Similarly, Leon and Dineen [165] reported that injection of a virus (48 h and 72 h prior to heat exposure) exacerbated EHS severity and survival in mice during recovery, even after sickness behaviors had extinguished.

Considered together, the above observations suggest that the existence of endotoxemia or infection, before and during strenuous exercise-heat exposure, increases the risk of EHS [121]. These findings also suggest that optimizing the intestinal barrier and immune system defenses may reduce the risk of EHS morbidity and mortality. Indeed, one theory [66, 121] views the development of EHS along two paths: endotoxemia and heat toxicity. In the former, EHS is viewed as an illness which originates within the gut, when GI temperature is 42°C or less, as the rate of LPS translocation into blood overwhelms the rate of LPS clearance by Kupffer cells in the liver. The resulting endotoxemia leads to systemic hypotension, cardiovascular shock, and disseminated intravascular coagulation [44–47], sometimes existing without hyperthermia. This interesting concept may explain why some cases of EHS occur in cool or mild environments [41, 161, 164, 166]. In the second theoretical pathway [66], severe hyperthermia above ~42°C causes damage to the cytoskeleton and other cellular structures and eventually leads to multiorgan necrosis and death [43, 74, 76]. The authors of this theory believe that the former path (i.e., endotoxemia) transitions to the latter (i.e., hyperthermia) but that no information exists regarding the mechanism of this transition [121]. This distinction may have greater academic than medical value because, in clinical practice, EHS therapy involves immediate aggressive whole-body cooling, transport to an emergency care facility, treatment of signs and symptoms, and monitoring of laboratory tests during recovery [2, 3, 167]. In severe EHS cases, both endotoxemia and hyperthermia act in concert to induce multiorgan dysfunction and damage.

### 5.9. Aging

Plasma levels of LPS are elevated in older adults [168, 169], and they may experience chronically increased inflammatory tone, apparent as increased concentrations of the inflammatory markers TNF-a, IL-6, IL-8, and C-reactive protein [16, 170]. Reduced microbial diversity and stability [16, 171], as well as changes in the predominant bacterial phyla, also may occur. Williamson et al. [172] reported that adults older than 74 years experienced a 10-fold increase of blood infections with* Escherichia coli*, Gram-negative bacteria, when compared to adults aged less than 50 years. Likewise, Klevens and colleagues [173] observed that older (versus younger) adults experienced an increased incidence of blood infections with* Staphylococcus aureus*, Gram-positive bacteria. This may occur because elderly subjects exhibit a prolonged inflammatory response (i.e., TNF-*α*, and IL-10), relative to younger participants, when hospitalized with a pneumococcal infection [174]. Only a small number of publications have focused on the influence of aging on intestinal permeability, and no research studies have evaluated age-related differences of inflammatory tone in EHS patients. However it is widely recognized that older adults experience EHS and nonexertional heatstroke more commonly than their younger counterparts, as reported in multiyear studies involving Japanese citizens [175], and Muslims participating in the desert pilgrimage to Mecca [176]. This latter study described 125 EHS cases, showing that incidence rose precipitously across the age groups 40–49, 50–59, and 60–69 years; 58% of these EHS patients were 60 years or older. This phenomenon may be related to decreased cardiovascular physical fitness and heat intolerance in the elderly, but that was not the case in a report involving South Africa laborers [177], where the age threshold for an increased incidence of EHS in deep mines was >40 years. Also, preexisting comorbidities (e.g., hypertension, diabetes) may affect thermoregulation, heat tolerance [178], and systemic inflammatory tone.

### 5.10. Nonsteroidal Anti-Inflammatory Drugs

Ironically, evidence suggests that it is unwise to use a nonsteroidal anti-inflammatory drug (NSAID) to reduce pain, fever, and inflammation, prophylactically or after exercise. Short-term and prolonged moderate-intensity or low-intensity aerobic exercise, when combined with aspirin or ibuprofen use, may increase gastrointestinal permeability and the risk of endotoxemia [46, 179–181]. A controlled field study involving 29 ultramarathoners provided evidence of this effect [182]. Runners consumed 600 mg of ibuprofen on the day before, and 1200 mg on the day of, an arduous 160 km endurance event in a cool-to-moderate environment. When compared to 25 control runners (no NSAID intake), those who ingested ibuprofen experienced significantly higher (*P* = 0.04) LPS and proinflammatory cytokine (IL-6, IL-8, and IL-10) concentrations in plasma. Interestingly, no between-group differences were observed in race time, gastrointestinal discomfort, perceived muscle soreness, or muscle damage biomarkers in plasma. Human investigations, conducted at rest in mild environments, also have demonstrated that NSAID increase plasma cytokine concentrations. When given i.v. LPS (*Escherichia coli *endotoxin), participants developed fever (rise of 1.3°C) and illness symptoms including chills, myalgia, headache, and nausea at 1 h that were diminished by 5 h after injection. A 4- to 10-fold increase of TNF-*α*, and a 10-fold increase of IL-6, occurred 1.5–3 h after an 800 mg ibuprofen pretreatment and subsequent challenge with i.v. LPS; the control experiments involved i.v. normal saline prior to the LPS challenge [183, 184]. The peak levels of TNF-*α* (i.e., a primary mediator of septic shock and organ failure) were considerably higher than the levels commonly observed in patients with sepsis [184]. The mechanism is unknown, but two hypotheses are relevant to intestinal ultrastructure and function [185]. First, NSAID may reduce prostaglandin production in the intestinal mucosa, resulting in damage via oxygen free radical production and enterocyte microvascular vasoconstriction [186]. Second, NSAID may reduce cellular ATP production by inhibiting energy production at specific steps in the glycolytic and tricarboxylic acid cycle pathways [187]. Together, these actions may affect intestinal epithelial cells in a way that orchestrates failure of energy-dependent intracellular mechanisms, which regulate the integrity of tight junctions [188]. Also, it is likely that several other medications are absorbed by increasing intestinal permeability, via the action of tight junctions [189, 190]. These research findings are disturbing, considering the widespread use of NSAID by endurance athletes. During the 2004 New Zealand Ironman triathlon, for example, 30% of competitors consumed NSAID during the race [191], whereas, during the 2008 Brazil Ironman event, 43% of triathletes who were surveyed consumed NSAID one day before, 68% immediately before, and 89% during the race [192]. A study of runners at the 1996 Chicago marathon also reported that 75% of subjects ingested aspirin or ibuprofen before or during the race [193].

### 5.11. Prescription Medications and Drugs of Abuse

Pharmacologic agents have been recognized for more than 50 years as predisposing or causative factors for nonexertional heatstroke, exertional heatstroke, and drug-induced hyperthermic conditions [17, 51, 120, 194, 195]. The classes of these drugs include anticholinergics, antihistamines, monoamine oxidase inhibitors, amphetamines and their analogues, diuretics, antihypertensives, antipsychotics, and antidepressants [43, 51, 196, 197], plus inhalation anesthetics which affect individuals who have a genetic susceptibility for malignant hyperthermia [198, 199]. Because each drug has unique pharmacologic effect(s) on cells, tissues, and organs [200] and because human thermoregulation is complex, it is impossible to reduce these effects to a single mechanism. However, authors have attributed fatal hyperthermia to numerous effects, including increased metabolic heat production; diminished sweat production or skin blood flow; interference with hypothalamic temperature regulation or cardiac output; and fluid-electrolyte imbalances [44, 51, 74, 132, 165, 194, 196, 201, 202].

Overdoses of psychostimulant drugs such as cocaine, amphetamine, methamphetamine, and ecstasy (i.e., 3,4-methylenedioxymethamphetamine, MDMA) are of particular interest because hyperthermia is common in severe or lethal poisonings and may be the primary mode of death [196, 203, 204]. Even though their central and peripheral effects are unique, these drugs share a final common pathway with EHS: stimulation of the sympathetic nervous system and increased heat production [195, 204]. In cases of EHS [44–47] and in cases of drug overdose, with or without hyperthermia [205–207], disseminated intravascular coagulation, rhabdomyolysis, and liver injury occur. Indeed, severe EHS and cocaine intoxication share many clinical signs and symptoms [4]: combative behavior, coma, nonpyrogenic hyperthermia, acute renal failure, oliguria, hyperuricemia, rhabdomyolysis, hypotension, seizure, myoglobinuria, hypocalcemia, disseminated intravascular coagulation, multiorgan hemorrhage, and hepatic damage (i.e., elevated plasma ALT, AST, and bilirubin). These commonalities suggest similar etiologies for EHS and cocaine intoxication, and that the primary suspected noxious insults are hyperthermia and oxidative damage in tissues. The mechanisms responsible for the oxidative damage induced by hyperthermia are not fully understood, but it is known that they include formation of reactive oxygen species (e.g., superoxide and hydroxyl radicals) in liver tissues and that the resultant oxidative stress mediates heat-induced cellular damage [208, 209].

Because oxidative stress has been implicated in neurodegenerative diseases, aging, cancer, and vascular diseases, elimination of unwanted reactive oxygen species is very important [210]. In controlled laboratory investigations of organ damage via overdoses of acetaminophen [211, 212], cocaine [213], MDMA [214], amphetamine [215], and morphine [216, 217], oxygen free radicals played an important role in hepatotoxicity [218]. Two additional studies support this concept. First, vitamin E (i.e., an inherent antioxidant defense against toxic free radical species) deficiency increased susceptibility to MDMA-induced neurotoxicity and hepatic necrosis [219]. Second, when mice were treated with morphine plus two exogenous antioxidants (i.e., glutathione and ascorbic acid), the damaging effects of morphine on liver cells was completely abolished [217]. However, it is difficult to identify the exact pathways by which drugs induce acute and chronic liver damage, because various pathways of cell death are triggered, with significant crosstalk and overlap. These involve disruption of hepatocyte ultrastructures (e.g., membrane, nucleus, and cytoplasm) and processes (e.g., gene translation, RNA cleavage) [220].

Both LPS and the proinflammatory cytokines (i.e., TNF-*α*, IL-1) mediate hepatotoxicity in response to a variety of chemicals (e.g., carbon tetrachloride, acetaminophen, *α*-naphthylisothiocyanate, alcohol, and galactosamine) [221–224]. In one relevant, controlled laboratory investigation, Meng et al. [225] observed two groups of mice: those treated with morphine (pellet implantation, 24 h) and a control group (placebo pellet, 24 h). Plasma levels of morphine were in the range experienced by opioid abusers and patients taking opioids for moderate-to-severe pain. Employing a variety of techniques, researchers determined that morphine disrupted the tight junctions which connect small intestine epithelial cells, increased gut permeability, allowed bacterial translocation (i.e., plate incubation) to the liver and lymph nodes surrounding the intestine, and fostered enterocyte tissue inflammation (i.e., microscopic examination of histological sections). Other rodent studies have determined that (a) chronic morphine use accelerates the progression of LPS-induced sepsis to septic shock [226]; (b) both chronic morphine and morphine withdrawal lower host immune defense against IM bacteria (reviewed in [225]); and (c) the opioid antagonist naltrexone (i.e., a prescription drug used in the management of alcohol and opioid dependence) blocks acute endotoxic shock in mice by inhibiting TNF-*α* production [227]. These studies have implications for individuals and governmental health care, because morphine and other opioid drugs are the most widely prescribed and abused pain medications in the United States.

## 6. Advanced Planning and Education Reduce EHS Morbidity and Mortality

In most cases of EHS, exercise induces hyperthermia due to a situation (e.g., sport competition, work site, or military activities) that pushes a healthy, young athlete, laborer, or soldier to the point of collapse and medical emergency [51]. Knowing this, attempts to reduce EHS morbidity and mortality begin with awareness and prevention. First, laborers and athletes who perform strenuous work or exercise in warm or hot environments (a) must understand the nature and predisposing factors of EHS (Table 1); (b) recognize the early warning signs of EHS (i.e., dizziness, headache, loss of coordination, great fatigue, and nausea; Table 6); (c) limit exercise/work intensity and duration to their present level of physical fitness and heat acclimatization; and (d) consume adequate fluids. Second, supervisors (i.e., race organizers, medical directors, foremen, and military officers) must modify exercise-rest or work-rest cycles in real time by monitoring environmental conditions [2]; recognize that central nervous system involvement (e.g., loss of mental acuity, confusion, and aberrant behavior) is a hallmark sign of EHS, a medical emergency [3]; and prepare cold water, ice, and large immersion tubs as emergency treatment for EHS [167, 228]. Although EHS cannot be predicted, hyperthermic death and circulatory shock can be avoided in virtually all cases, with early recognition and rapid immersion in cold or ice water [2, 3, 229, 230]. In addition to conductive cooling, water immersion supports the central circulation via external hydrostatic pressure and skin vasoconstriction.

## 7. Do Foods or Nutritional Supplements Augment the Intestinal Barrier and Immune Function?

Preexisting baseline endotoxemia or viral infections are believed to exacerbate hypercoagulation, the production of proinflammatory cytokines, and organ injury in cases of EHS [50, 147, 165]. Recognizing plasma LPS level as a measurable and critical factor in the development of EHS, the preceding text describes several intrinsic characteristics and behaviors that increase intestinal permeability, thereby increasing the risk of severe EHS. The following paragraphs consider foods and nutritional supplements that (a) have anti-inflammatory or antioxidant effects and (b) facilitate effective immune responses. The effects are accomplished via influences on IM homeostasis, intestinal permeability, liver function, or immune defenses.

### 7.1. Probiotic Supplements

The number of bacteria administered with a daily probiotic dose, in capsule or tablet form, is approximately 10^9^ or 10^10^ cells. In comparison, the complex human IM bionetwork is 1,000–10,000 times larger (10^13^ organisms). Thus, in terms of sheer numbers, it is somewhat surprising that probiotics remain in the gut or exert measurable effects on physiology, metabolism, and immunity [231, 232]. Also, in terms of intestinal secretions, probiotic bacteria also must be resistant to gastric acidity and bile salts (Table 3). Research evidence suggests that this is possible, in that* Bifidobacterium longum* can persist in the gut of 30% of individuals for at least 6 months after oral consumption, without causing gastrointestinal symptoms or altering the species composition of the resident microbiota [233]; this should be interpreted in view of the fact that longevity may not be characteristic of other species [234, 235].

The functional targets of probiotics include electrolytes and water absorption, lactose digestion/absorption, mucosal integrity, and epithelial proliferation and repair [236]. To our knowledge, there is little evidence to suggest that people with a normal IM ecosystem can benefit from probiotic consumption [231]. However, substantial evidence suggests that oral probiotic use imparts positive effects on a variety of human illnesses and chronic diseases, including gastroenteritis, lactose intolerance [237], infectious diarrhea in children [238], antibiotic-associated diarrhea,* Helicobacter pylori*,* Clostridium difficile* disease [239], and incidence and mortality due to necrotizing enterocolitis in premature infants [240]). Probiotics may or may not alleviate some of the symptoms of irritable bowel syndrome, a condition for which the current support is weak [239, 241]. Negative effects of administering probiotics directly into the intestine [242] have been reported among patients hospitalized with severe pancreatitis (probiotics group, *n* = 152; placebo group, *n* = 144). This randomized, double-blinded protocol did not reduce the risk of infectious complications and was associated with an increased risk of mortality (probiotics group, 16%; placebo group, 6%; relative risk, 2.53). The authors speculated as to the cause of these deaths in critically ill patients, mentioning the administration of 10 billion probiotic bacteria per day, plus enteral nutrition, in the same region of the intestine. This intervention might have aggravated local inflammation of the intestinal mucosa, reduced capillary blood flow to enterocytes, and induced ischemia [242].

As noted above, EHS involves disrupted intestinal barrier integrity due to hyperthermia (Figure 3), decreased splanchnic blood flow, and enterocyte hypoxia. Relevant to EHS and metabolic endotoxemia, several controlled investigations have evaluated the influence of probiotic consumption on intestinal barrier integrity, with positive outcomes. These studies indicate that probiotics may initiate repair of damaged epithelial cells by redistributing tight junction proteins and restoring the mucosal lining which surrounds enterocytes [234, 243–246]. It also is well established that probiotic bacteria can suppress intestinal inflammation [234] by downregulating LPS receptors on epithelial membranes, secreting metabolites (Table 2), and/or inhibiting cell signaling in humans and in mice [247, 248]. Lamprecht and colleagues [249] reported similar findings during a randomized, double-blinded, placebo-controlled study that compared a probiotic containing six bacterial strains (*n* = 11) to a placebo (*n* = 12), during a 14-week treatment phase. They reported that probiotic supplementation improved intestinal barrier function and low-grade inflammation in men under sustained exercise stress (80–90 minutes of strenuous exercise). Several other single-strain probiotics are reported to provide beneficial effects on intestinal barrier function, by modifying expression and localization of tight junction proteins in the space between enterocytes [249]. Unfortunately, hundreds of bacterial species exist in the human IM ([8]; Table 2, Figure 1), probiotic effects are likely to be strain specific [234, 235, 250], and targeted prophylactic benefits are difficult to validate scientifically in controlled, randomized trials.

### 7.2. Anti-Inflammatory and Antioxidant Foods

Diet, by providing the substrates for bacterial metabolism, can modulate inflammation and immune function, pH of the mucosal lining surrounding intestinal epithelial cells, intestinal permeability, gut hormone release, glucose homeostasis, insulin secretion, and energy metabolism [103, 133, 135, 251] and regulate epithelial permeability by modifying expression and localization of tight junction proteins in the paracellular space [252]. Low-grade plasma LPS increases adipose proinflammatory cytokine release (e.g., TNF-*α* and IL-6) and may contribute to insulin resistance [146]. Also, the composition and biological functions of the IM (Figure 2) are different in humans who consume vegan, vegetarian, and omnivorous diets, as well as those high in red meat [253, 254]. Thus, we propose that habitual dietary choices may predispose individuals to EHS because of a preexisting increase of plasma LPS and low-grade inflammation, and human food consumption patterns are reflected in both the IM and its collective genome [12, 255, 256]. Our proposal is supported by a 2013 porcine investigation which evaluated the influence of different dietary oils on intestinal LPS transport and serum endotoxemia, following a single meal [257]. Four groups of animals consumed one meal of 500 g ground corn-soybean meal dough; this was mixed with either 50 ml water (control), 50 ml fish oil, 50 ml vegetable oil, or 50 ml coconut oil (one per group). Relative to the control condition, postprandial serum LPS concentration (and a measure of intestinal permeability to LPS) increased after a meal rich in saturated fatty acids (coconut oil) but decreased after a meal containing higher omega-3 fatty acids (e.g., cod liver and fish oils).

Changing the composition of one's diet rapidly shifts the microbial ecosystem [253, 258–260]. This effect was observed within 24 hours, during a controlled-feeding study of 10 adults who initiated a high-fat/low-fiber or low-fat/high-fiber diet [256]. Similarly, a shift of the IM was observed within 24 h, during both phases of a study involving a plant-based diet (i.e., rich in grains, legumes, fruits, and vegetables) and an animal-based diet (i.e., predominantly meats, eggs, and cheeses).* Bacteroides* was highly associated with animal protein, a variety of amino acids, and saturated fats;* Prevotella*, in contrast, was associated with high intakes of carbohydrates and simple sugars [253]; both are Gram-negative anaerobic bacteria. These effects are modest and reversible and vary among individuals [14, 129, 253, 254].

Virtually all bacteria in the colon digest plant materials via fermentation [129]. The resulting metabolic products (Table 2) include short-chain fatty acids (SCFA) [130, 131, 133]. The SCFA (e.g., butyrate, acetate, and propionate) are generated by Gram-negative and Gram-positive bacteria [131, 261], mostly by fermentation of dietary carbohydrates that humans cannot digest (i.e., nonstarch polysaccharides, resistant starch, and dietary fiber) [129]. Butyrate is an important energy source for intestinal columnar epithelial cells (i.e., preventing mucosal degradation) [262] and influences tight junction protein expression [101]. Relevant to EHS, SCFA in general influence colon and liver blood flow, encourage colon mucosal integrity [102], and modulate both plasma LPS [134] and cytokine responses [130, 133].  Table 7 presents foods that have moderate-to-high anti-inflammatory and antioxidant qualities. A diet that is rich in vegetables, fruit, nuts, and fish theoretically encourages colonic health by minimizing inflammation and counteracting oxygen free radicals [263–268]. In support of this concept, Ghanim and colleagues [140] reported that a single high-fat, high-carbohydrate meal (i.e., 41% carbohydrate, 17% protein, and 42% fat) increased plasma LPS (+47%) and elevated production of reactive oxygen species (+65 to 78%) for 2-3 hours; a meal rich in fiber and fruit (e.g., 58% carbohydrates, 15% protein, and 27% fat), as recommended by the American Heart Association, induced no significant change of either LPS or reactive oxygen species.

Athletes are quite interested in the potential ergogenic effects of foods and nutritional supplements, to the point that they purposefully alter their diets. These manipulations include carbohydrate loading across several days (i.e., to increase stored glycogen deposits in skeletal muscle and liver tissues), protein supplementation across weeks and months (i.e., to encourage muscle hypertrophy, strength, and recovery, concurrent with heavy resistance training), chronically consuming low-carbohydrate diets (i.e., to induce production of ketones as a fuel during endurance exercise), and weight loss diets which allow athletes to “make weight” in sports with body weight classes [269–274]. Unfortunately, these specialized diets may be low in fiber, nonstarch polysaccharides and nondigestible starch [101, 253, 275], which anaerobic bacteria convert into SCFA in the colon. As an example, an animal-based diet (e.g., meats, eggs, and cheeses) decreases the abundance of bacteria in the phylum Firmicutes (Table 2, Figure 1) which metabolize dietary plant polysaccharides [253, 254] and decreases levels of SCFA (i.e., acetate and butyrate) in the gut, compared to a plant-based diet (e.g., grains, legumes, fruits, and vegetables) [258]. Theoretically, this suggests that intestinal functions may be compromised, in ways that are important to exercise performance, including IM effects on colon and liver blood flow, carbohydrate and lipid absorption, energy metabolism, enzyme expression, insulin sensitivity, free fatty acid oxidation, and electrolyte-water absorption in the colon [101–103]. In our opinion, the unknown effects of carbohydrate loading, protein supplementation, and low-carbohydrate diets on the IM, intestinal permeability, and plasma LPS levels are worthy of future investigation.

### 7.3. Antioxidant Supplements

Although a small amount of gastrointestinal permeability and low basal levels of plasma LPS are normal, and a healthy immune system prevents adverse effects, low levels of endotoxin are rapidly removed by inflammatory cells in the liver (Kupffer, macrophages, and neutrophils) [276]. Based on published review articles [277, 278]), research evidence does not support consuming antioxidant supplements when plasma LPS levels are normal. When circulating LPS levels are low, perhaps the only individuals who benefit from antioxidant supplements are those whose habitual diets are antioxidant-deficient. This may be due to the fact that uptake of antioxidant vitamins from supplements is likely to be influenced by the consumption of foods and when one's diet contains adequate antioxidants (Table 7), the circulation and tissues become saturated [277].

Endurance athletes have elevated plasma LPS antibody (anti-LPS IgG) concentrations at rest [84–86, 88] due to repeated, small exposures to LPS that has translocated from the gut during prolonged exercise [88, 94, 96, 97]. Plasma LPS concentration rises when the liver is unable to cope with a large influx of LPS from the intestine; at a greatly exaggerated level, this also occurs in severe EHS cases that involve systemic hypotension, cardiovascular shock, and liver injury [41–47]. In these cases, hepatocytes and Kupffer cells are exposed to LPS, proinflammatory cytokine release is stimulated (TNF-*α*, IL-6), and biochemical reactions generate oxygen free radicals, which have been implicated in various types of acute and chronic liver injury [276]. Free radical oxidative damage occurs in proteins, lipids, cell membranes, mitochondria, and DNA; antioxidant compounds (Table 7) are chemical reducing agents that inhibit the oxidation of other molecules by being oxidized themselves.

Scientists understand much about the complex antioxidant systems and enzymes which plants and animals maintain [279, 280]. However, science has not resolved an interesting paradox regarding antioxidant supplements. Specifically, research evidence from controlled, randomized trials in humans and animals suggest that vitamin E (*α*-tocopherol), vitamin A (*β*-carotene), and lycopene supplements provide positive antioxidant effects that diminish biomarkers of liver injury [276, 281–283]. These findings suggest, in theory, that antioxidant supplementation encourages efficient liver function, or reduce free radical damage in some disease states. In healthy individuals, large-scale clinical trials (i.e., randomized, double-blind, and placebo-controlled) and meta-analyses of antioxidant supplements have shown either no effect [284, 285] or harmful effects such as an increased risk of prostate cancer [286], hemorrhagic stroke [287], first nonfatal myocardial infarction [288], and mortality [289]. Although several theoretical explanations for this paradox exist [290–292], it is clear that additional research is required.

### 7.4. Amino Acid Supplements

No pharmacologic agent is known to successfully treat fulminant EHS. Amino acid supplements, however, offer a prophylactic approach for possibly preventing/reducing hyperthermia-induced or exercise-induced disruption of the intestinal barrier [293]. Via unknown mechanisms, supplemental glutamine treatment has been shown to reduce or prevent heat-induced intestinal permeability in mice [294], rats [295], and humans [296]. It is possible that glutamine influences this change of permeability by serving as an important fuel source for intestinal enterocytes. Moreover, intestinal permeability was inhibited by pretreatment with arginine supplementation, in mice exercising in a hot environment to a rectal temperature of 39.6°C [297]. Possible protective mechanisms have been proposed, including that glutamine and/or arginine activate immune function, inhibit oxidative stress, stimulate heat shock protein expression, or influence the structure of epithelial cell tight junctions (i.e., ultrastructural bridges that allow LPS translocation from the intestine to plasma) [293–295]. Further, it is possible that L-arginine's role as a precursor to nitric oxide synthesis influences intestinal permeability by modifying blood flow to enterocytes.

### 7.5. Effects of Diet on Immune Function

Luminal and epithelial immune cells recognize bacterial LPS fragments in the intestine [30]. Because diet influences these immune responses [298, 299], a number of review articles have focused on the negative effects of nutrient deficiencies, malnutrition, and body weight loss [300–302], as well as nutrients that are required for effective immune responses. These essential nutrients include amino acids, vitamins (e.g., A, folic acid, B_6_, B_12_, C, and E), minerals (e.g., zinc, copper, iron, and selenium) [300], omega-3 polyunsaturated fatty acid in fish oils, and a favorable ratio of omega-3 to omega-6 fatty acids [299, 303, 304]. Further, the reader is reminded that SCFA are products of the fermentation of dietary fiber and complex plant polysaccharides which humans cannot digest (see above). The levels of SCFA in the colon and in blood are important to immunoregulation because they stimulate immune function and resolve inflammation [299]. The concentration of SCFA also affect translocation of microbes [298] and subsequent intensity of immune responses, by enhancing barrier function [265] and decreasing intestinal permeability. Thus, a diet which contains ample vegetables, fruits, and nuts produces important immunomodulatory products.

Prolonged or intense exercise presents unique systemic and cellular demands on the immune system that far exceed those of daily living, in two ways. First, both intense and prolonged exercise bring about a proportional increase in plasma stress hormones (i.e., cortisol, epinephrine, and norepinephrine), which induce changes in immune function (e.g., decreased white blood cell count and increased levels of pro- and anti-inflammatory cytokines) [301, 302]. Second, the increased oxygen free radical formation, which accompanies the dramatic rise of mitochondrial oxidative metabolism during strenuous exercise, potentially could inhibit neutrophil, lymphocyte, and natural killer cell responses [305, 306]. These unique demands suggest that athletes, laborers, or soldiers may require greater quantities of vitamins and minerals with immune-enhancing properties. Unfortunately, the roles of reactive oxygen species during prolonged or intense exercise are unclear, and the endogenous antioxidant defenses in the human body are complex, interlocking, and carefully regulated [290]. Further, the body's “total antioxidant capacity” seems unresponsive to high doses of dietary antioxidants, so that the amount of oxidative damage to key biomolecules is rarely altered [291]. Given the lack of randomized, placebo-controlled, blinded studies, we encourage researchers to investigate these matters in future human studies that involve dietary influences on immune function, during exercise-heat exposure.

## 8. Summary

The following factors may alter the IM ecosystem or increase intestinal permeability, plasma LPS, and inflammation: preexisting illness or fever [50, 161, 162], gastroenteritis [161], poor cardiovascular physical fitness [17, 22], lack of heat acclimatization [94, 97], sleep deprivation [116–118, 164], prescription medications and drugs of abuse [196, 203, 204], alcoholism [155, 156], NSAID use [46, 179–182], and dehydration [122]. Although all of these factors have been recognized previously as predisposing factors for EHS (Table 1), the unique contribution of the present manuscript is the evidence-based focus on their relationships to the IM, endotoxemia, immune function, oxygen free radical injury to tissues, and diet.

Beginning with the premise that restoring or preserving intestinal integrity (i.e., blocking the transfer of LPS from the intestine to general circulation) is a logical step in preventing or attenuating tissue/organ injuries [307], we recommend specific foods that have anti-inflammatory and antioxidant properties. A diet that contains ample vegetables, fruits, and nuts produces important immunomodulatory products which intestinal bacteria metabolize. This dietary plan agrees with the position of the American Dietetic Association [308], the recommendations of the United Nations and World Health Organization [309], and research findings [310, 311]. Although the theoretical connections among these factors are strong, definitive human studies do not exist [263, 268] because the influences of the IM and diet on EHS were not research priorities in years past. We now encourage future research that assesses the IM ecosystem, habitual dietary patterns, plasma LPS, immune function, oxygen free radicals, and biomarkers of inflammation among EHS patients and control subjects. This research should include the purposeful dietary manipulations of athletes, such as carbohydrate loading, protein supplementation, and ketogenic diets which may encourage dysbiosis in the IM ecosystem.

We realize that this research will be complicated by the large interindividual variation of responses (i.e., to exercise-heat stress, diet, and LPS) [29, 312–314]; the human genome [129, 315–318]; the vast, dynamic IM genome [9, 15, 127, 319]; temporal changes of the IM ecosystem in response to numerous environmental and lifestyle factors (e.g., antibiotics, disease, exercise; [13, 320]); and the fast-acting influence of a single dose of LPS on intestinal permeability [321]. Our incomplete understanding of these complex interactions explains, in part, why EHS cannot be predicted or prevented at present.

## Figures and Tables

**Table 1:** Predisposing factors for exertional heatstroke.

Hot, humid environment with direct solar radiation and little wind movement
Insulated clothing
Low ratio of skin surface area to body mass
Recent (1–3 days) stressful heat exposure
Lack of heat acclimatization
Medications that alter sweat gland function, cutaneous vasoconstriction, or cardiac contractility
Skin disorders (e.g., ectodermal dysplasia, burns, anhidrosis)
Strenuous or prolonged exercise
Overweight, obesity
Dehydration
Diarrhea, vomiting
Use of diuretics and laxatives
Fever and illness
Infectious disease
Low cardiorespiratory physical fitness
Alcohol abuse or drug use
Older age
Sleep deprivation

*Sources*. [1–3, 50, 164, 322].

**Table 2:** Human intestinal microbiota: phyla, metabolism, and potential biological functions.

Intestinal segment	Predominant bacterial phyla^a^	Bacterial metabolism		Potential biological functions of bacteria metabolites
		Biochemical substrates^b^	Metabolic intermediates & products^b^	
Small intestine	Firmicutes Bacteroidetes Proteobacteria^c^ Actinobacteria	Resistant starches, dietary fiber (cellulose, pectin, inulin), carbohydrates, amino acids, lipids, triglycerides, carboxylic acids, creatine, pancreatic/gastrointestinal secretions, and mucus.	Complex carbohydrates, amino acids, lactic acid, ethanol, succinic acid, and formate produce short-chain fatty acids^d^ (SCFA; acetate, propionate, butyrate), branched-chain fatty acids (BCFA), CO_2_, H_2_, NH_3_, H_2_S, amines, phenols, biotin, and vitamins K, B_1_, B_2_, B_6_, B_12_.	Absorb fats and lipid-soluble vitamins, stimulate systemic hormones to regulate lipid and glucose homeostasis, energy regulation, modulate pro- and anti-inflammatory genes, strengthen epithelial permeability, regulate colon pH, inhibit pathogen growth, provide energy to luminal epithelium.
Large intestine^e^	Bacteroidetes, Firmicutes, Proteobacteria^c^			

*Notes*. ^a^Inter-individual differences are great; ^b^composition of substrates, intermediates and products change along the course of the intestinal tract; ^c^the outer membrane of *Proteobacteria* is composed mostly of LPS; ^d^most SCFA exist in higher concentrations in the proximal (versus distal) colon; ^e^phyla differ markedly in proximal versus distal colon, in part due to differences in oxygen tension. Abbreviations: IM, intestinal microbiota; LPS, lipopolysaccharide. Sources: [135, 152, 154, 253, 319, 323–327].

**Table 3:** Characteristics of the human intestine and resident microbiota.

Intestine segment	Intestine functions	Intestine anatomy, circulation, and environment	Bacterial characteristics^a^	Microbe load per g of luminal contents
Small intestine (1–3 cm diameter; length, duodenum 0.4 m, jejunum 2.5 m, ileum 3.5 m)	Absorbs > 95% of essential dietary nutrients (e.g., water, ions, amino acids, peptides, glucose, fructose, lipids, iron, vitamins). Peristalsis mixes and moves chyme distally. Secretions neutralize stomach acid and chemically digest food (e.g., bile salts). Endocrine feedback blocks stomach acid production and stimulates pancreatic insulin secretion. Supports local immunity against pathogens.	Surface of the luminal epithelium is covered with microvilli, which contain digestive enzymes and membrane nutrient transporters. Aerobic environment with pH of 6-7. Partial pressure of oxygen (*p*O_2_) in terminal ileum tissue, 33 mmHg. Blood supplied by superior mesenteric artery. At rest, intestinal capillaries receive ~20% of total cardiac output, but ~75% of these capillaries are not perfused when unfed (i.e., without glucose or other nutrients).	Aerobic species and facultative anaerobes (with bimodal metabolism) predominate. Gene activity analysis indicates: amino acids > carbohydrates > cofactors/vitamins > lipid metabolism.	Duodenum and jejunum, <10^3^; Ileum, 10^3^–10^7^.

Large intestine (6 cm diameter; length, cecum 0.2 m, colon 1.4 m, rectum 0.1 m)	Absorbs unabsorbed water and vitamins. Secretions neutralize acidic pH. Compacts waste for elimination. Rectum stores feces temporarily. Supports local immunity against pathogens.	Microvilli are absent. Anaerobic environment (*p*O_2_ of sigmoid colon tissue, 3 mmHg; rectum, <1 mm Hg) with a pH of 5–7. The lumen center has a *p*O_2_ < 0.1 mmHg.	Caecum has greatest diversity (500–1,000 species); facultative anaerobes dominate. Distal colon has smallest species diversity; obligate anaerobes dominate.	Colon, 10^9^–10^12^; Feces, 10^10^–10^12^

^a^Facultative anaerobes grow with or without oxygen; obligate anaerobes grow only in the complete absence of oxygen and process substrates via fermentation; both Gram-positive and Gram-negative bacteria populate the small and large intestine segments. Sources: [152, 259, 325, 328–330].

**Table 4:** Potential effects of the intestinal microbiota (IM) and dietary intake on immune function^a^.

Intestinal lumen sites/processes	Bacterial effects on immune function	Influence of diet
Mucus layer^b^	(i) Block cell adhesion sites of pathogenic bacteria	
	(ii) Site of competition for nutrients with pathogenic bacteria	X
	(iii) Affect function of mucosal immune cells/factors^a^	X
	(iv) Alter bacterial diversity and abundance of specific phyla	X

Epithelial cell layer	(i) Goblet cells secrete mucins (high molecular weight glycoproteins) which prevent entry of pathogens and noxious substances	
	(ii) Paneth cells contain antimicrobial peptides	
	(iii) Express heat shock proteins which reduce inflammation and injury^c^	X
	(iv) Influence the function of lymphocytes, leukocytes (T-cells, macrophages, secretory immunoglobulin A), neutrophils, monocytes^a^	X

Biochemical/metabolic processes	(i) Produce antimicrobial substances that inhibit pathogenic bacteria	
	(ii) Produce LPS, which affects systemic, liver, adipose tissue inflammation	X
	(iii) Modulate antibody production in response to large antigen load	X
	(iv) Degrade toxins and toxin receptors	
	(v) Bacterial metabolites (e.g., SCFA) have pro- and anti-inflammatory effects	X

^a^Due to its vast surface area, the human intestine houses the largest number of immune cells in the human body. ^b^Mucus layer is the first line of defense against physical and chemical injury caused by ingested food, microbes, and microbial products; ^c^heat shock protein expression increases. IM, intestinal microbiota; LPS, lipopolysaccharide, an endotoxin; SCFA, short-chain fatty acids. Sources: [52, 103, 130, 133, 154, 316, 327, 330–333].

**Table 5:** Potential interactions among intestine anatomy/physiology, bacteria, immune function, diet, exercise stress, and the host genome as predisposing factors for exertional heatstroke (EHS).

	Hypothetical involvement in EHS
Intestine anatomy & physiology	(i) Gut anatomy (i.e., crypts) and the surrounding mucus layer facilitate immune homeostasis, protects commensal species from bacterial competitors, and reseeds the IM after the ecosystem has been altered/depleted [331, 334]. (ii) Epithelial membrane integral proteins (i.e., toll-like receptors) recognize bacteria and other microorganisms. Once activated, these receptors can recruit immune cells and produce cytokines, which in turn regulates the number and diversity of bacteria in the gut [335]. (iii) Disruption of normal bowel function as a result of infection or inflammation uncovers its critical importance for acid-base homeostasis and normal mucosal pH [336]. (iv) Hyperthermia damages membranes of intestinal epithelial cells [31], disrupts tight junctions [337], and increases permeability to LPS^a^ [32, 101]. This permeability change occurs at temperatures of 41.5–42.0°C when sustained for 60 min [31]. (v) Human nonexertional heatstroke patients (mean rectal temperature, 42.1°C) exhibit increased plasma LPS [68, 69]. (vi) The epithelial mucosa becomes acidic during intense, anaerobic exercise [73, 88, 180, 338]. (vii) Hypoxia in the intestinal mucosa releases highly reactive oxygen and nitrogen species that accelerate mucosal injury [45, 339]; similar hypoxia-induced production of ROS and RNS occurs in liver cells [44].

Bacteria	(i) Products of bacterial metabolism (a) increase intestinal permeability and plasma LPS concentration, (b) strengthen the epithelial cell barrier, and (c) modulate expression of both proinflammatory and anti-inflammatory genes [135]. Bacterial metabolic products influence both innate and adaptive immune cell functions [340]. (ii) Commensal bacteria produce short-chain fatty acids (which have anti-inflammatory properties in multiple immune cell types); they also synthesize vitamins and amino acids which influence immune function [130, 135]. (iii) In patients with chronic inflammatory bowel diseases (e.g., Crohn's disease, ulcerative colitis) and alcoholic liver disease, the IM differs from control subjects, and plasma LPS is chronically elevated [152, 155, 244, 330]. (iv) An array of diseases and dysfunctions (e.g., atherosclerosis, burn injury) have been hypothetically associated with an imbalance of the composition, numbers, or habitat of the IM [11, 154, 330]. (v) Several bacterial activities have been linked to increased risk of gastric and colorectal cancer [154, 330].

Immune function	(i) The IM can modulate innate and adaptive immune responses at mucosal surfaces during infection, inflammation, and autoimmunity [170]. (ii) Changes in the crosstalk between the intestinal epithelium, the intestinal immune system, and gut microbes modulate systemic immunity [335]. (iii) LPS is released upon the death of Gram-negative bacteria. LPS is a potent stimulus for the release of cytokines. The resulting inflammatory response can alter thermoregulation and result in multiple-organ dysfunction [42]. LPS can cause death at plasma concentrations as low as 1 ng/mL [43]. (iv) Proinflammatory (TNF-*α*, IL-1*β*) and anti-inflammatory (IL-6, IL-10) cytokine concentrations in plasma are elevated during exercise-induced hyperthermia and exertional heatstroke [44]. (v) Severe EHS victims may succumb to a condition similar to sepsis [41–43], mediated by leakage of LPS from the intestinal lumen into the circulation. This leads to an immune (i.e., cytokine) inflammatory response culminating in systemic hypotension, cardiovascular shock, and multiple organ failure [44–46]. EHS fatalities among primates exhibit greater coagulopathy, inflammation, and tissue injury than hyperthermic survivors [42]. (vi) LPS also stimulates blood coagulation; thus EHS-induced microthrombosis and hemorrhage occur in tissues of the intestine, liver, lungs, kidneys, pancreas, spleen, skin, cornea, heart, brain, and adrenals [43, 49–51]. (vii) Administration of immunomodulators, antibodies to endotoxin, and corticosteroids improve survival in animals with heatstroke and attenuate hemodynamic instability, but have not been studied in humans [44]. (viii) At rest, pre-hydration with an intravenous glucose-NaCl solution shifts cytokine (TNF-*α*, IL-1*β*, IL-8) responses to injected human endotoxin towards a more anti-inflammatory balance [123].

Diet	(i) Diet modulates inflammation and immune function at rest [103, 133] (ii) At rest, diet modulates the pH of colon mucosa, intestinal permeability, as well as glucose, insulin, and energy metabolism [135]. (iii) A change of diet rapidly alters IM composition [253, 256]. (iv) A high-fat, low-fiber Western diet promotes the overgrowth of gram-negative pathogens, with consequent increased intestinal translocation of bacterial LPS [153]. (v) Obesity and Type 2 diabetes are associated with a chronic low-grade inflammatory state, known as “metabolic endotoxemia” [341, 342] because these diseases involve translocation of LPS from the intestinal lumen into blood. Extensive research involving mice demonstrated that a 4-week high-fat diet chronically increased plasma LPS levels [153], and induced obesity and insulin resistance. Altering the IM of mice by antibiotic administration protected mice from fat mass development, glucose intolerance, insulin resistance, mild endotoxemia, inflammation [136]. (vi) Metabolic endotoxemia (interrelationships between LPS, a high-fat diet, obesity, and Type 2 diabetes) has been confirmed in multiple studies involving healthy and obese humans (publications reviewed by [40]).

Exercise stress (intensity & duration)	(i) Numerous studies have reported lower splanchnic and mesenteric blood flows during strenuous exercise; this can result in hypoxia, intestinal barrier disruption [34, 122, 180, 343]. This hypoxic state, evident in the intestinal villi and lobes of the liver, likely results in ATP depletion, acidosis, and altered membrane ion pump activity [43, 51, 339, 344]. (ii) EHS and non-exertional (classical) heatstroke often involve systemic acidosis [51, 345, 346]. (iii) During high intensity (80% VO_2peak_; [87]) and prolonged (>9 h; [84, 86]) exercise, the incidence of endotoxemia (plasma LPS) increases. (iv) Exercise-induced (running 60 min at 70% VO_2max_) mild dehydration (−1.5% body mass loss) increases intestinal permeability [122]. (v) Efficient energy metabolism (i.e., biochemical generation of ATP) is essential during prolonged or intense exercise. Bacteria may influence energy metabolism by modulating intestinal transit time (energy harvest); polysaccharide degradation to monosaccharides; glucose absorption into intestinal epithelial cells; de novo lipid production; FFA and glucose oxidation in liver, muscle, and adipose tissue [103]. (vi) Exercise relies on the uptake of glucose by skeletal muscle, mediated by insulin that is produced in the pancreas. In some adults (e.g., those with Type 2 diabetes), skeletal muscle and liver tissues exhibit resistance to the action of insulin. (vii) The total amount of energy available to a cell is limited. Exercise and high body temperature cause Na^+^-K^+^-ATP_ase_ pumps to operate at a high rate. Eventually, cells can become energy depleted, they swell due to reduced water transport (implying a reduced pump activity), and rigor mortis sets in, implying energy depletion [34, 76]

IM, intestinal microbiota; LPS, lipopolysaccharide; TNF-*α*, tumor necrosis factor alpha; IL-1*β*, interleukin-1*β*; IL-6, interleukin-6; IL-10, interleukin-10; ROS, reactive oxygen species; RNS, reactive nitrogen species; FFA, free fatty acid; ^a^a major component of the cell wall of Gram-negative bacteria.

**Table 6:** Clinical and laboratory results, which are observed in advanced cases of exertional heatstroke and exemplify multiple organ dysfunction or failure.

Signs and symptoms	Laboratory/autopsy findings
(i) Internal body temperature > 40°C (ii) Hyperventilation (iii) Headache (iv) Central nervous system involvement (loss of mental acuity, fatigue, weakness, confusion, dizziness, delirium, loss of coordination) (v) Hypotension (vi) Tachycardia (vii) Circulatory shock (viii) Liver failure (ix) Kidney failure (x) Nausea, vomiting (xi) Diarrhea	(i) Intestinal ischemia (ii) Metabolic acidosis with respiratory alkalosis (iii) Elevated plasma lactate (iv) Elevated plasma LPS (v) Elevated plasma cytokines (vi) Elevated hematocrit (vii) Elevated liver enzymes (ALT, AST) in plasma (viii) Disseminated intravascular coagulation (ix) Leukocytosis (x) Neutropenia (xi) Tissue hemorrhage with necrosis (xii) Intestinal lesions

*Sources*. [43, 44, 119, 120].

**Table 7:** Foods which have moderate-to-strong anti-inflammatory^a,c^  and antioxidant^b,c^  effects.

Characteristics	Representative foods	Active constituents^d^	References^d^
Anti-inflammatory	*Vegetables*: avocado, cabbage, carrot, chili pepper, kale, onion, spinach, *Fruit*: apple, blueberry, cherry, grape, grapefruit, orange, strawberry, watermelon *Nut*: almond, Brazil nuts, cashews, hazelnut, macadamia, peanut, pine nut, pistachio, pecan, walnut *Fish*: anchovy, herring, mackerel, salmon, sardine *Other*: black pepper,clove, caraway, extra virgin olive oil, flaxseed, green coffee, licorice, nutmeg, oregano, wine (red), sage, tea, thyme	DHA, EPA, capsaicin, carotenoids, curcumin, flavonoids, maresins, monosaturated fats, monoterpenes, omega-3 fatty acids, polyphenols, protectins, quercetin, resolvins, resveratrol, sulfides	Kang et al., 2009; Landberg et al., 2011; Mueller et al., 2010; Sears, 2015 [303]; Tsai et al., 2005; Wu & Schauss, 2012 [267], Bolling et al., 2011 [263]; Watzl, 2008

Antioxidant	*Vegetables*: beet, beet root, black carrots, cauliflower, chili pepper, garlic, ginger, leek, lettuce, potato, red pepper, turnip, yam *Fruit:* black berry, cherry, strawberry *Nut*: pecan *Other*: mint, rice bran, turmeric	*α*-tocopherol, *β*-carotene, anthocyanins, carotenoids, ellagitannins, flavonoids, lycopene, phytoestrogens, polyphenols, proanthocyanidins, sulfides	Kaur & Kapoor, 2002; Tsai et al., 2005; Wu et al., 2004 [347]; Hassan & Abdel-Aziz, 2010; Bolling et al., 2011 [263]; Watzl, 2008; Gülçin, 2012

^a^Typically determined via blood tests for proinflammatory cytokines (IL-6, TNF*α*, and IL-1), chemokines, acute-phase proteins, cell adhesion molecules, and adipokines [267]; ^b^determined via laboratory analyses of the antioxidant activity of food constituents, including phenolic compounds (e.g., flavonoids, isoflavones, and proanthocyanidins), antioxidant vitamins (*α*-tocopherol, *β*-carotene), lipids (carotenoids, sterols), total antioxidant capacity (lipophilic plus hydrophilic methods), and/or the oxygen radical absorbance capacity assay [263, 266, 282, 347]. ^c^Multianalyte profile technology identifies inflammatory and oxidative biomarkers simultaneously in serum or plasma [348]; ^d^also see the US Department of Agriculture database at https://ndb.nal.usda.gov/ndb/. DHA, docosahexaenoic acid; EPA, eicosapentaenoic acid.
